# Anisotropic Phonon Bands in H-Bonded Molecular Crystals: The Instructive Case of α-Quinacridone

**DOI:** 10.1021/acsmaterialsau.3c00011

**Published:** 2023-05-26

**Authors:** Lukas Legenstein, Lukas Reicht, Tomas Kamencek, Egbert Zojer

**Affiliations:** Institute of Solid State Physics, NAWI Graz, Graz University of Technology, Petersgasse 16, 8010 Graz, Austria

**Keywords:** phonons, quinacridone, hydrogen bonding, organic semiconductor, vibrations, molecular crystals

## Abstract

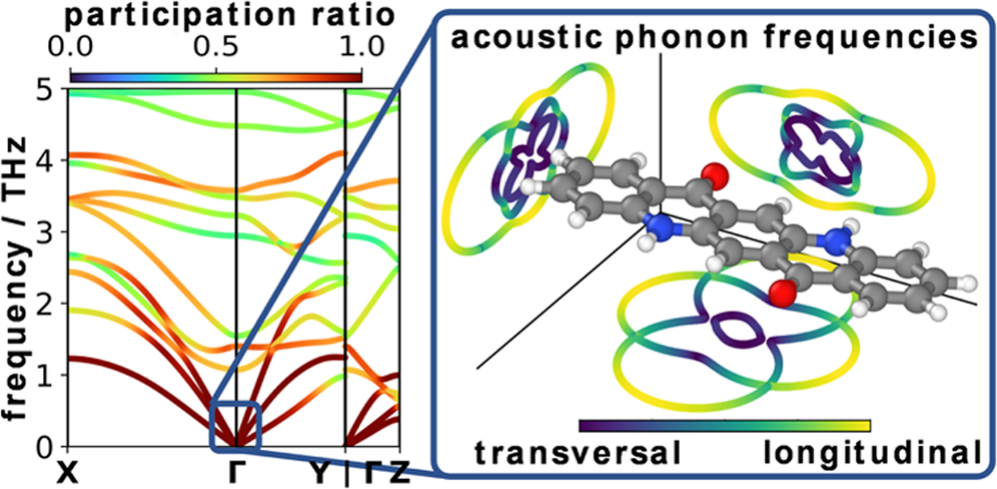

Phonons play a crucial role in the thermodynamic and transport properties of solid materials. Nevertheless, rather little is known about phonons in organic semiconductors. Thus, we employ highly reliable quantum mechanical calculations for studying the phonons in the α-polymorph of quinacridone. This material is particularly interesting, as it has highly anisotropic properties with distinctly different bonding types (H-bonding, π-stacking, and dispersion interactions) in different spatial directions. By calculating the overlaps of modes in molecular quinacridone and the α-polymorph, we associate Γ-point phonons with molecular vibrations to get a first impression of the impact of the crystalline environment. The situation becomes considerably more complex when analyzing phonons in the entire 1st Brillouin zone, where, due to the low symmetry of α-quinacridone, a multitude of avoided band crossings occur. At these, the character of the phonon modes typically switches, as can be inferred from mode participation ratios and mode longitudinalities. Notably, avoided crossings are observed not only as a function of the length but also as a function of the direction of the phonon wave vector. Analyzing these avoided crossings reveals how it is possible that the highest frequency acoustic band is always the one with the largest longitudinality, although longitudinal phonons in different crystalline directions are characterized by fundamentally different molecular displacements. The multiple avoided crossings also give rise to a particularly complex angular dependence of the group velocities, but combining the insights from the various studied quantities still allows drawing general conclusions, e.g., on the relative energetics of longitudinal vs transverse deformations (i.e., compressions and expansions vs slips of neighboring molecules). They also reveal how phonon transport in α-quinacridone is impacted by the reinforcing H-bonds and by π-stacking interactions (resulting from a complex superposition of van der Waals, charge penetration, and exchange repulsion).

## 1 Introduction

Hydrogen-bonded molecular crystals are appealing because hydrogen bonding improves the supramolecular ordering and promotes self-assembled growth.^1^ This eases the control of parameters like molecular orientation.^2,3^ Quinacridone (5,12-dihydroquinolino[2,3-*b*]acridine-7,14-dione), as a prototypical hydrogen-bonded organic semiconductor, has a long history of industrial application as an organic dye in pigment violet 19.^4^ Beyond that, there are various proposed device applications^5^ that make use of quinacridone’s (QA) electrical and optical properties. These include organic light-emitting diodes,^6,7^ field-effect transistors,^8^ organic solar cells,^9^ and photothermal evaporators.^10^

The application of hydrogen-bonded organic semiconductors (OSCs) in organic devices has been accompanied by a thorough experimental and theoretical investigation of their electronic and optical properties.^11,12^ In contrast, studies of the phonon properties of such materials have so far been limited to the measurement and simulation of vibrational spectra.^13,14^ These solely probe the Brillouin zone center (Γ-point) and fail to reveal the often complex dispersion relations of phonons in H-bonded crystalline materials. Phonon properties of the entire 1st Brillouin zone, however, play a major role for quantities like the thermodynamic stability,^15^ the heat capacity, and all transport properties. Phonons (from within the entire 1st Brillouin zone) are the main carriers of heat in molecular crystals^16,17^ and, therefore, dictate the thermal conductivities of OSCs, which typically differ between directions within and perpendicular to the planes in which the OSC molecules are arranged.^18−21^ Additionally, in OSCs, the coupling between charge carriers and phonons is particularly strong, thus significantly inhibiting charge carrier transport,^22−24^ where in recent studies, the dominant role of specific phonon modes has been identified.^25−28^ In the context of charge transport, it has also been shown that a proper description of electron–phonon couplings in OSCs requires the consideration of phonons from the entire 1st Brillouin zone.^29^ Measuring the phonon band structures of molecular crystals is, however, quite challenging, as it typically requires large single crystals of fully deuterated materials.^30^ In fact, to the best of our knowledge, to date, only the phonon band structures (simultaneously characterizing phonon frequencies and wave vectors) of deuterated naphthalene, anthracene, and perylene have been measured with inelastic neutron scattering.^30−32^ For more complex and/or nondeuterated organic materials, phonon densities of states (i.e., phonon spectra in which the wave vector dependence is not resolved) have been determined.^25,28,33−35^ More recently, inelastic X-ray scattering has been applied to determine parts of the dispersion of rubrene crystals, albeit still combined with a tremendous experimental effort.^36^ As an alternative approach, simulating phonons of OSCs employing state-of-the-art dispersion-corrected density functional theory provides accurate Γ-point phonon frequencies.^25,37−41^ Even more importantly, for the aforementioned deuterated naphthalene, they also accurately reproduce the measured phonon band structures.^15,39,42^ Simulations have the additional advantage that they provide access to quantities beyond phonon frequencies and their dispersion relation. These include, for example, vibrational eigenvectors, which allow an in-depth analysis of the phonon properties, as will be exploited below.

For the present study, we, thus, simulated the phonon properties of α-polymorph of quinacridone (α-QA) from first-principles by calculating the atomic force constants from ab initio forces within the harmonic approximation with phonopy’s^43^ finite difference scheme.^44^ The (very minor) impact of anharmonicities is discussed in the Methods Section and in much more detail in the Supporting Information. Our focus is on gaining a fundamental understanding of phonons in the low-frequency region (below 6 THz or 200 cm^–1^), which is of primary importance for heat transport.^45^ In this region, also the rigid translation and rotation modes with relatively strong coupling to electrons are found.^25^

For a study of the fundamental phonon properties of OSCs, α-QA is an ideal candidate due to the highly anisotropic bonding interactions in this material. Moreover, the molecular stacking motive in α-QA is comparably simple: it contains only one molecule in the unit cell with molecules arranged in stripes. These stripes are close to parallel to the molecular planes and form layers, which adopt a slipped π-stacking arrangement (see the next section). Consequently, one can identify directions with predominantly H-bonding, van der Waals (vdW), and π-stacking interactions. These directions are essentially parallel to the short and long molecular axes and perpendicular to the molecular planes. In passing we note that attractive dispersion forces play a dominant role in all directions, while π-stacking besides attractive charge penetration^46^ primarily triggers exchange repulsion, as discussed in detail in ref (47). Nevertheless, we will stick to the above terminology (H-bonding, π-stacking, and vdW bonding directions) throughout the entire manuscript.

The possibility to reliably assign certain spatial directions to specific interaction mechanisms makes α-QA an ideal candidate for a fundamental analysis of the anisotropic coupling between molecules. This would not be possible for molecules in a herringbone arrangement, which is common for OSCs, or for the β-polymorph of QA,^48^ where neighboring QA stripes run in different directions. For the electronic band structures, the particularly instructive situation in α-QA has already been exploited for studying peculiarities of the anisotropic electronic coupling between molecules.^12,47^ Here, we will study the impact of the α-QA structure on vibrational properties and the phonon band structure: first, we will trace the vibrations in the crystalline phase back to those of molecular QA, which explains how the molecular eigenmodes are modified by the crystalline environment. An in-depth analysis of the phonon band structure of α-QA then allows identifying how band dispersions (and, thus, phonon group velocities) are impacted by different types of bonding interactions. The discussion of the angular dependence of phonon frequencies (i.e., of angular band structures) then shows how vibrational eigenmodes change as a function of the direction of phonon propagation and what role avoided band crossings play in this context. This, finally, allows an analysis of the evolutions of the group velocities of low-frequency modes in general and acoustic phonons in particular. The latter are of distinct importance, as they crucially determine the thermal transport properties of the material.

## 2 Structure of α-QA

**Figure 1:**
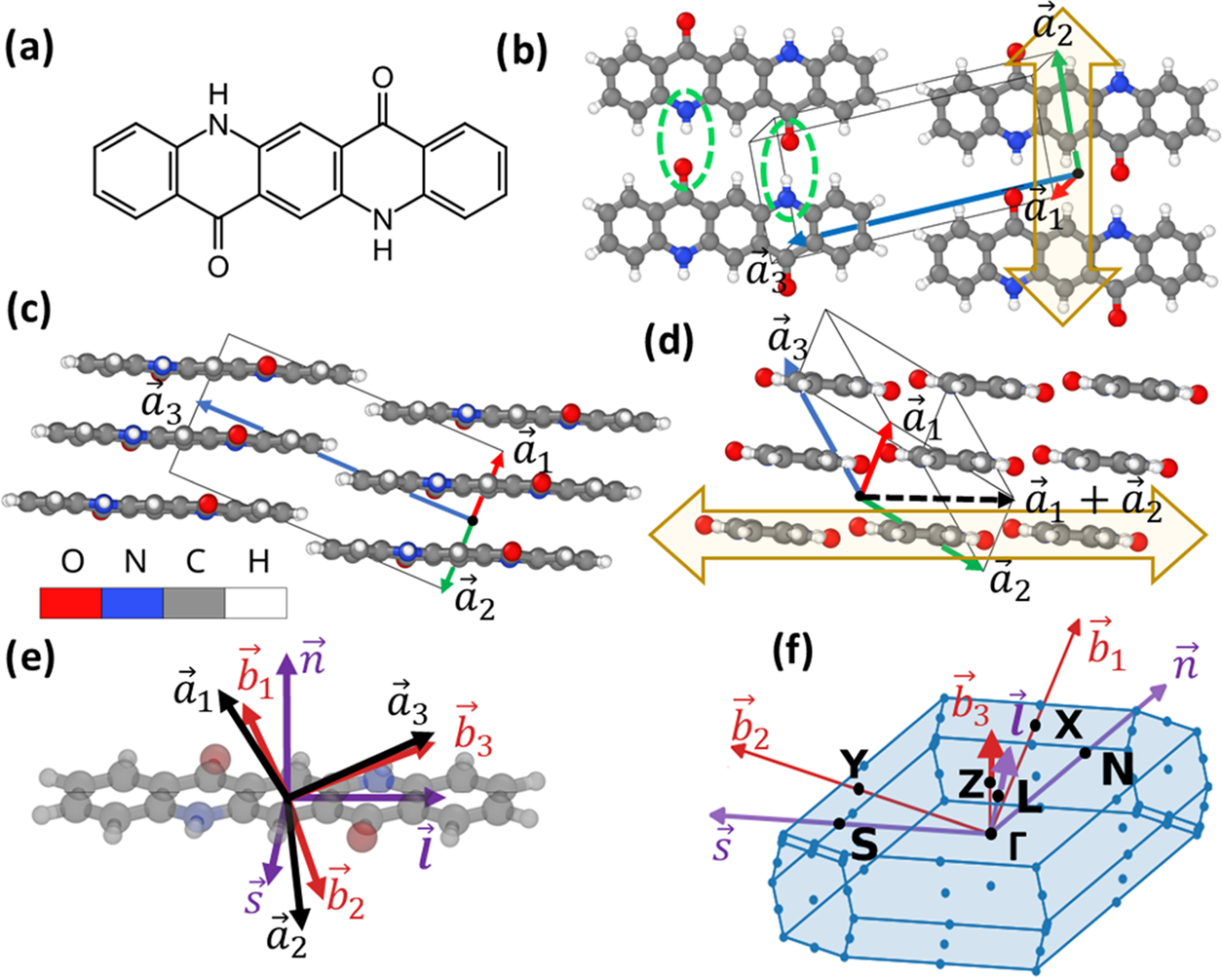
Panel (a) shows the chemical structure of an isolated QA molecule, while panels (b)–(d) illustrate the molecular packing in the crystalline α-polymorph viewed in the directions perpendicular to the π-plane, along the short molecular axis, and along the long molecular axis, respectively. The lattice vectors *a⃗*_1_, *a⃗*_2_, and *a⃗*_3_ and the unit cell are shown in panels (b)–(d), and the atoms are colored using the code shown in the inset of panel (c). The green ellipses in panel (b) denote the atoms partaking in the H-bonding and the transparent yellow arrows in panels (b) and (d) highlight the directions of the H-bonded QA stripes. The relation between lattice vectors (black arrows), reciprocal lattice vectors (red arrows), the direction normal to the molecular plane *n⃗*, the short, and the long molecular axes *s⃗* and *l⃗* is shown in panel (e). The 1st Brillouin zone and the intersection with the molecular axes (purple) and the reciprocal lattice vectors (red) are illustrated in panel (f). The intersection points are labeled; they are the points referred to in Figures 4a, 5, and 6.

As shown in Figure 1a, in the gas phase, the intramolecular conjugation between the benzene rings of QA is disturbed by the amine and carbonyl groups. This is largely overcome in the solid state through the formation of H-bonds between the molecules, which results in a particularly large reduction of the band gap.^49^ In α-QA, the molecules are arranged in one-dimensional (1D) stripes (transparent yellow arrows in Figure 1b,d) with two H-bonds to each of the two neighboring molecules (green ellipses in Figure 1b). The centers of neighboring molecules within the stripes are connected by the *a⃗*_1_ + *a⃗*_2_ vector (with *a⃗*_1_ and *a⃗*_2_ being two of the real-space unit cell vectors; see Figure 1). As shown in Figure 1d, the short molecular axis is slightly inclined relative to the direction of the stripes. Nevertheless, the stripes as well as the short molecular axes are essentially aligned with the H-bonding direction in the α-QA crystal. The H-bonded stripes adopt a slipped π-stacking arrangement, with the slip between neighboring QA stripes amounting to 1.52 Å along the long and 0.98 Å along the short molecular axes. This results in a distance of 3.44 Å between the π-planes. In the *a⃗*_3_ direction, the molecules are tightly packed, with the close contact between hydrogens at the molecular ends leading to an interlocking of the molecules (see Figure 1c). This hinders translations of the QA layers in directions parallel to the *a⃗*_1_, *a⃗*_2_ plane. Figure 1e shows the relation between the crystal axes (black arrows), the reciprocal lattice vectors (red arrows), and the QA molecule, with its axes explicitly shown by the purple arrows. These molecular axes, the reciprocal lattice vectors, and their respective intersecting points with the boundaries of the 1st Brillouin zone are shown in Figure 1f and will be referred to when discussing phonon band structures in Section 4.

## 3 Methods

The crystal structure of the α-polymorph of QA^48^ was obtained from the Cambridge Crystallographic Data Center.^50^ Starting from the experimental structure, the atomic positions and lattice vectors were relaxed employing periodic boundary conditions until the maximum residual force component fell below 10^–3^ eV/Å. For this, the ab initio materials simulation package FHI-aims^51^ (versions “201103” and “220829”) was used, employing the Perdew–Burke–Ernzerhof functional (PBE)^52^ in combination with the nonlocal many-body dispersion correction by Herman et al. (MBD-NL). This combination has been shown to accurately describe a large variety of molecular complexes and crystals, including systems with intermolecular O···H–N bonding.^53^ For the geometry optimizations, a trust radius method enhanced BFGS algorithm was applied.^54,55^ The interatomic force constants needed to derive phonon properties were obtained from finite atomic displacements using a symmetry-assisted finite difference scheme employing a modified version of the Parlinski–Li–Kawazoe method,^44^ as implemented in the phonopy package (v.2.9.3).^43^ To obtain a converged electronic structure of α-QA, reciprocal space was sampled by a 6 × 4 × 2 Γ-centered *k*-point grid in combination with default FHI-aims “tight” basis sets for each atomic species (for more details, see the Supporting Information). Converged phonon band structures in α-QA with the above settings require the considerations of 4 × 3 × 2 supercells (amounting to lattice vector lengths of 15.4, 19.3, and 29.3 Å), which after applying all symmetries resulted in 108 single-point calculations on displaced structures. A test of the convergence of the supercell size can be found in the Supporting Information (Section 8). As amplitude for the atomic displacements for calculating the force constant and dynamical matrices, we chose the default value of 0.01 Å. Systematically varying the amplitude of this displacement between 0.001 and 0.02 Å resulted in negligible frequency shifts and eigenvector deviations, i.e., the force constants from these finite difference calculations yield identical vibrational properties. Also for displacements of 0.05 Å, only rather minor differences were observed. This suggests that anharmonic effects have only a minor impact on the calculated phonon frequencies especially at low temperatures. The impact of thermal expansion on the vibrational properties of α-QA is not considered here. Still, it should be mentioned that also when employing the experimental, room-temperature unit cell parameters instead of the optimized ones, no significant modifications of the vibrational properties of α-QA were observed. This is shown together with details on various convergence and (an)harmonicity tests in the Supporting Information.

In passing we note that the results of the above-described FHI-aims calculations employing the MBD-NL van der Waals correction are essentially equivalent to those calculated with the Vienna Ab initio Simulation Package,^56−58^ employing Grimme’s D3 correction with Becke Johnson damping^59^ (see the Supporting Information). Especially, the latter approach has been thoroughly benchmarked for molecular crystals in the past and is known to excellently reproduce experimental data in the low-frequency region.^15,41^

Phonon band structures, group velocities, eigenvectors, and eigenmode displacements were calculated from the force constant matrices either directly with the phonopy package or employing our own python scripts, which make use of the phonopy–python interface.^43^ Eigenmode characteristics and labels were identified by generating and analyzing animations of the eigenmode displacements with the Ovito visualization tool (version 3.7.11).^60^

The vibrational properties of an isolated QA molecule were calculated with FHI-aims again using the PBE functional and the MBD-NL van der Waals correction. Here, instead of employing open-boundary conditions, we opted for calculating the forces within the finite difference scheme for a single QA molecule in a (100 × 100 × 100) Å^3^ cell with periodic boundary conditions. The enormous cell size serves to avoid couplings between periodic replicas. This approach was chosen for purely technical reasons, as it allows employing, for the nominally isolated molecule, the phonopy package using the same finite difference scheme (including symmetry considerations) as for the crystalline system. Furthermore, this approach ensures a consistent determination of eigenmodes in the molecule and in the crystal, which was useful for the projections of eigenmodes described below. To ensure the accuracy of the periodic approach for molecular vibrations, the latter were also computed for an isolated molecule in a finite difference scheme with an amplitude of the displacement of 0.0025 Å (as implemented in the script “get_vibrations.py” provided in the FHI-aims utilities). The two approaches yield equivalent results (see the Supporting Information). Using the eigenmodes obtained from the periodic approach, we calculated the eigenvector overlap between vibrations of QA in the gas phase and in the crystalline α-polymorph. The overlap elements σ_μν_ between vibrational mode μ in the gas phase and mode ν in the crystalline phase are given by the sum of the scalar products of the corresponding atomic eigenvectors
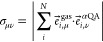
1

Here, *i* is an index that runs over all equivalent atoms, *N*, in the two systems and *e⃗*_*i*,μ_^gas^·*e⃗*_*i*,*ν*_^αQA^ is the scalar product of the corresponding atomic motions making up the eigenmodes. As the eigenvectors of gas phase vibrations and the vibrations in the crystals form complete bases, in which all α-QA eigenvectors can be described, one obtains  and . The overlap elements were then used to identify equivalent vibrations in the crystal and in the isolated molecules.

For a further analysis of the phonon bands of crystalline α-QA, a series of additional quantities were calculated. The mode participation ratio, *PR*_*ν*,*q⃗*_ (with band index *ν* and wave vector *q⃗*), quantifies to which degree all atoms of the structure participate in the vibrational motion of a particular eigenmode. Modes with high participation ratios involve the motion of large parts of the structure and represent “collective” oscillations of the atoms. Here, we refer to them as delocalized modes. Conversely, modes with small participation ratios comprise isolated vibrations of only one or a few atoms (e.g., N-H stretching) and are referred to as localized modes. Thus, the participation ratio is related to the “degree of delocalization” of a mode. Notably, in that spirit, delocalized and localized modes exist not only in molecular crystals but also in molecules, and the degree of localization is essentially unaffected by the fact that in a periodic structure, a certain group of atoms by virtue of translational symmetry will move in every unit cell. The participation ratio is defined as^61−63^
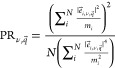
2where *N* refers to the number of atoms in the unit cell and *m*_*i*_ denotes the mass of atom *i*. As can be concluded from the definition, the participation ratio essentially measures, to what degree the mass-weighted atomic motions vary. The maximum value of unity (PR_*ν*,*q⃗*_ = 1) is obtained when all atoms within the unit cell perform the same motion. This, for example, occurs for pure translation modes, in which each atom of the molecule(s) oscillates with the same relative amplitude. The lower limit PR_*ν*,*q⃗*_ = 1/*N* is observed when only a single atom in the unit cell oscillates.

The “longitudinality” *L*_*ν*,*q⃗*_ of a mode is given by
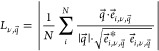
3

It measures for a specific mode *ν* at *q⃗*, how parallel the wave vector *q⃗* is to the atomic motions (associated with the eigenvectors *e⃗*_*i*,*ν*,*q⃗*_) of all *N* atoms in the unit cell. Note that both the wave vector and the eigenvectors are normalized and only their directions influence *L*_*ν*,*q⃗*_. As *q⃗* determines the momentum of the respective phonon, *L*_*ν*,*q⃗*_ = 1 identifies a fully longitudinally polarized (acoustic) mode, while fully transversally polarized (acoustic) modes are characterized by *L*_*ν*,*q⃗*_ = 0. By replacing the (mode-dependent) *q⃗* with a (fixed) vector parallel to a crystal or molecular axis, eq 3 can also be used to determine, to what extent a specific mode is polarized parallel to the chosen axis.

In general, group velocities are the derivatives of the phonon frequencies ω(*ν*, *q⃗*) (as the eigenvalues of the dynamical matrix) with respect to the wave vector *q⃗*
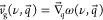
4

Considering that in simulations, reciprocal space is sampled in a discrete manner, the group velocities are here calculated from the expectation values of the *q⃗*-derivatives of the dynamical matrix *D*(*q⃗*) for the respective eigenvectors of *D*(*q⃗*), as implemented in phonopy^43^ via
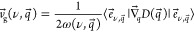
5

Sound velocities were obtained by calculating the group velocities in the long-wavelength limit for small wave vectors *q⃗* on a sphere with a constant radius |*q⃗*| = 0.0044 Å^–1^ (corresponding to 1% of the length of the shortest reciprocal vectors) around Γ.

For visualization purposes, the structure of α-QA and displacements of molecular vibrations and crystalline eigenmodes were plotted with Ovito,^60^ while for data analysis within Python, the libraries NumPy^64^ and SciPy^65^ were used. Phonon band structures and other vibrational properties were plotted using the Matplotlib^66^ libraries.

## 4 Results and Discussion

### 4.1 Molecular Vibrations in QA and Their Relation to Γ-Point Phonons in α-QA

**Figure 2:**
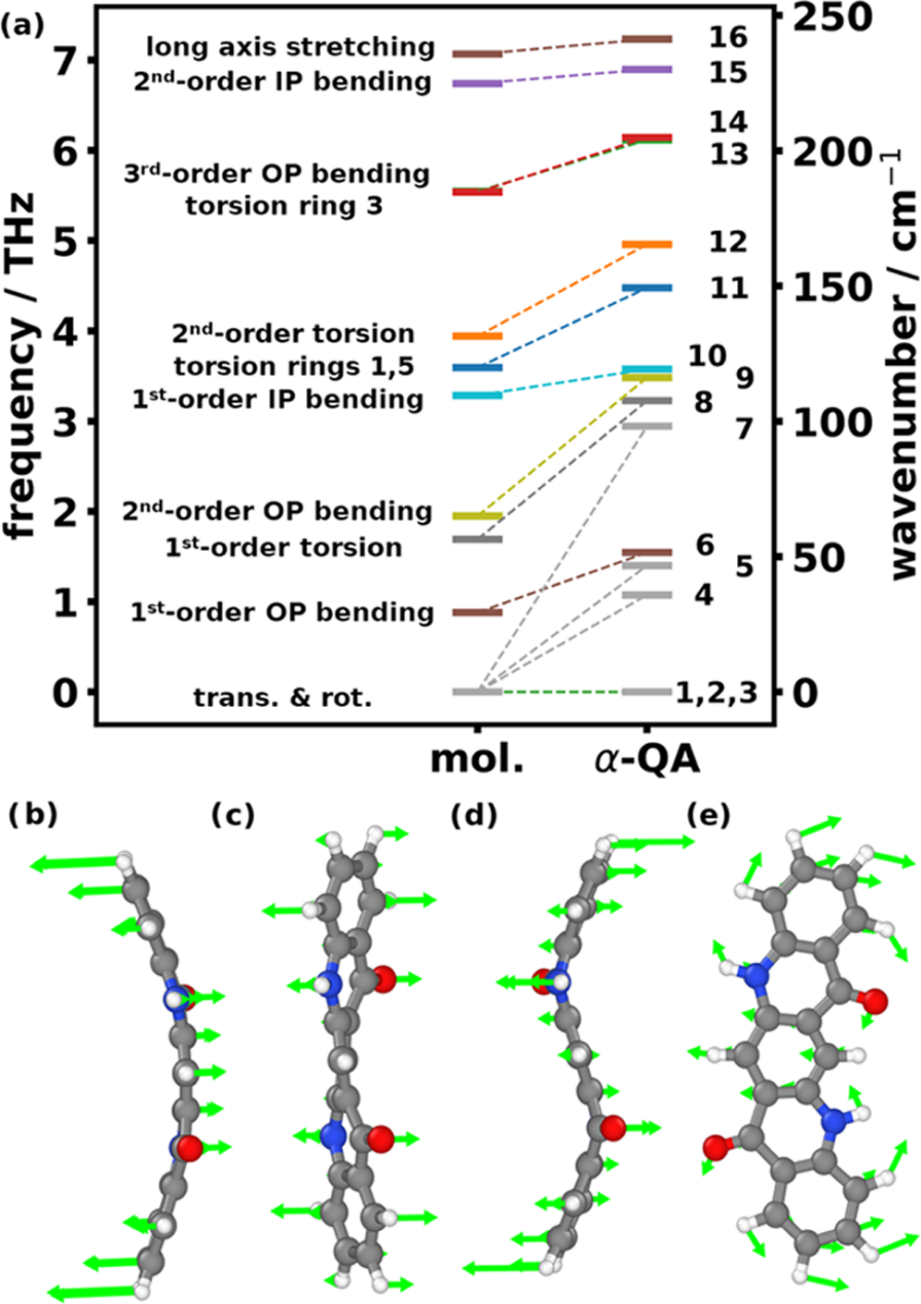
(a) Comparison between the low-frequency modes of the QA molecule and Γ-point frequencies in the α-QA crystal. (Largely) equivalent vibrations are connected by dashed lines, while details on the nature of the different types of vibrations can be found in the main text and are illustrated in panels (b)–(e): (b) 1st-order out-of-plane (OP) bending, (c) 1st-order torsion, (d) 2nd-order OP bending, and (e) 1st-order in-plane (IP) bending modes of molecular QA. The green arrows indicate (on a relative scale) how strongly the atoms are displaced when keeping the center of mass of the molecule fixed in space. The displacements of all eigenmodes contained in panel (a) can be seen in Figure S5 in the Supporting Information.

The vibrational eigenmodes of molecular crystals can be separated into intermolecular (external) and intramolecular (internal) modes. The former refer to motions of the molecules as rigid units and are determined by the arrangement of the molecules within the unit cell of the crystal. The latter are primarily comprised of deformations along the internal degrees of freedom of the molecule. Before analyzing the phonon bands in the crystalline α-phase, it is, therefore, useful to first consider the molecular vibrations of QA and to analyze how these eigenmodes are related to the intramolecular Γ-point modes of α-QA. Deviations from the molecular vibrations can then be associated with intermolecular interactions due to the different noncovalent forces at work in QA crystals. Regarding the intermolecular modes, the rigid translations of the QA molecule become the acoustic bands of α-QA, with vanishing frequency at the Γ-point. Conversely, the rotational molecular motions correspond to some of the lowest optical modes in the crystalline state. As shown in Figure 2, they are found in the same spectral region as the lowest four intramolecular modes. The latter are related to bending and torsion motions of the molecular backbones and their low frequencies can be traced back to the rather high flexibility of the comparably large QA molecules.

Here, bending mode refers to wave-shaped distortions of the molecular backbone, either along the normal to the molecular plane for out-of-plane (OP) modes (see, e.g., Figure 2b) or within the molecular plane for in-plane (IP) modes (see, e.g., Figure 2e). They are reminiscent of standing waves in a string and the number of nodes in the displacements increases with the order of the mode. This is illustrated for the 2nd-order OP bending mode in Figure 2d. Torsion modes comprise twisting motions of the rings around the long molecular axis. They can involve a torsional motion of the entire molecule like for the 1st-order torsion mode shown in Figure 2c, where the two halves of QA rotate in opposite directions. Alternatively, for certain modes, only individual rings are involved in the rotational motion, while the other rings hardly move at all (e.g., the ring-1–5 torsion, for which only the terminal rings rotate). The evolution of the frequency of such modes as a function of chain length has been discussed in ref (67) for the series of acenes based on analogies to simple classical oscillators. Finally, the last mode displayed in Figure 2a at 7.05 THz (235 cm^–1^) corresponds to a uniform, longitudinal stretching of the backbone. At (significantly) higher frequencies (not shown in Figure 2), we enter the realm of “classical” molecular vibrations commonly discussed in literature. These modes are often restricted to specific sections of the molecule or primarily concern the motion of specific chemical groups or atomic species (like C–H and C–C stretching and bending modes). Therefore, higher frequency modes already at the molecular level are usually characterized by rather low participation ratios, although up to ∼20 THz, there exist also modes in which the participation ratios reach values around 0.75, for example, for the cases of mode 27 at 14.09 THz (470.1 cm^–1^) and of mode 31 at 15.68 THz (529.1 cm^–1^) (see Figure S6 and Table S5 in the Supporting Information).

In the molecular crystal, the modes corresponding to rotations around the normal to the molecular plane, around the short molecular axis, and around the long molecular axis are eigenmodes 4, 5, and 7, respectively (eigenmodes 1–3 are the acoustic modes). Regarding the intramolecular modes, their order from the molecular system prevails also in the crystal at the Γ-point. For all vibrations displayed in Figure 2, the associated frequencies are shifted to higher values in the crystalline phase. Qualitatively, this can be explained by intermolecular interactions making the corresponding molecular deformations energetically more costly. Notably, such a shift to higher frequencies is observed also for nearly all higher frequency modes except for certain modes localized on atoms directly affected by the formation of the H-bonds (modes 89, 90, 107, 108; see Supporting Information Table S5). The particularly large shifts in the low-frequency region raise the question to what extent the vibrations in the crystal can be directly associated with molecular vibrations, i.e., to what extent the eigendisplacements in α-QA are comparable to those of the isolated molecule.

**Figure 3:**
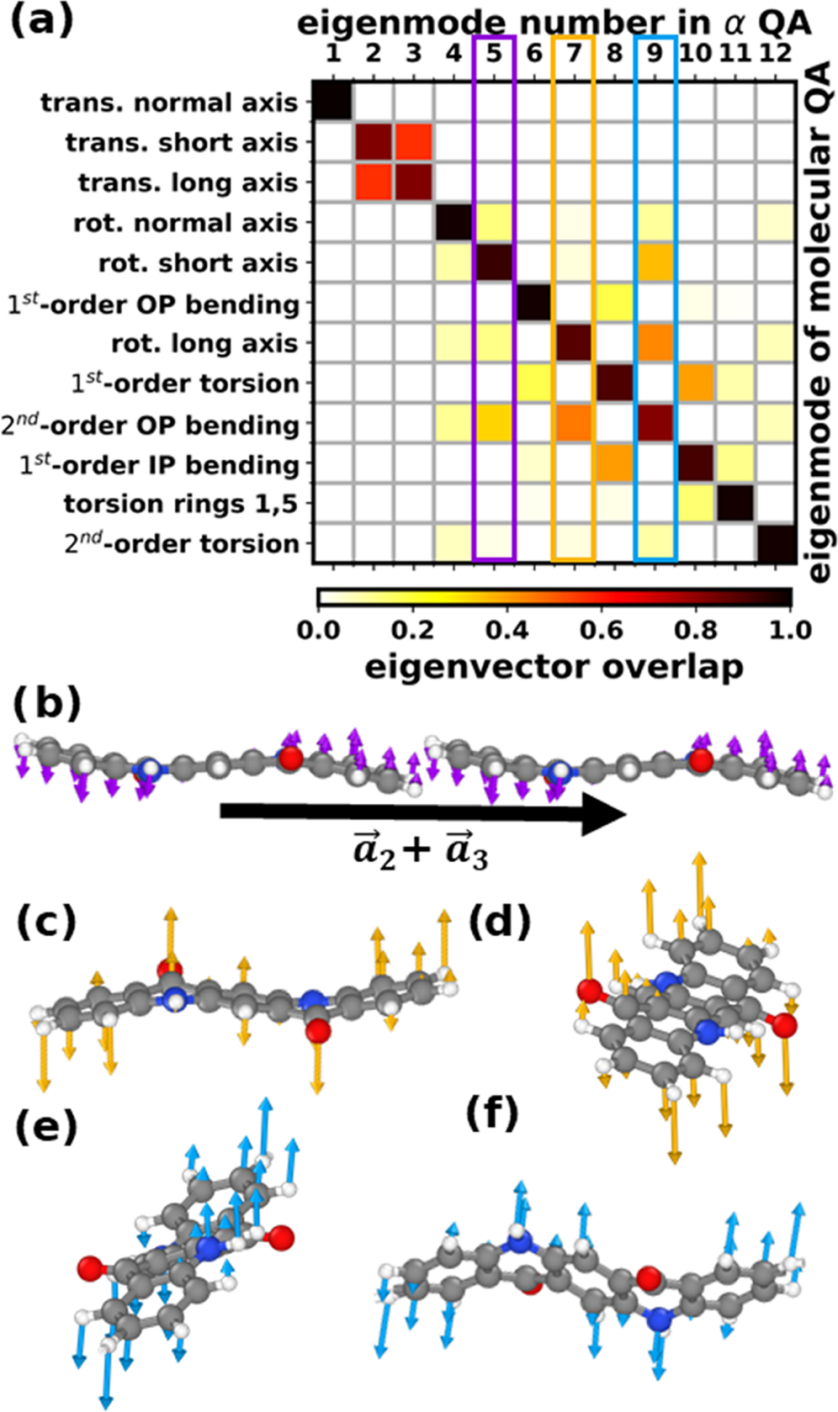
(a) Eigenvector overlap matrix of the first 12 molecular and α-QA vibrational modes. Values close to 1 imply strong similarity between molecular and crystalline modes, and elements below 1 imply hybridized modes. (b) Displacement pattern of mode 5 (purple) in α-QA relative to the equilibrium positions of the molecule and its periodic replica in the *a*_2_ + *a*_3_ direction. The arrows indicate the relative motion of the atoms; (c, d) pattern of atomic motions of mode 7 (yellow) and (e, f) of mode 9 (blue) in α-QA viewed from different directions.

This can be analyzed by calculating the eigenvector overlap matrix between the eigenmodes of α-QA and those of the isolated QA molecule employing eq 1. The resulting overlap matrix in Figure 3a shows how similar the lowest 12 modes of molecular QA and α-QA are (with a value of 1, denoting a perfect match; the actual values are listed also in Tables S5 and S6 in the Supporting Information). As mentioned above, the molecular translations form the basis of the acoustic phonons of α-QA. One of these acoustic modes in α-QA perfectly matches the molecular translation perpendicular to the plane of QA; the other two, at least at the Γ-point (where they are degenerate), turn out to be a superposition of the short-axis and long-axis translation of QA.

Hybridization effects also occur among the optical bands: for example, mode 5 is dominated by a short-axis rotation (σ = 0.93), which is mixed with a molecular 2nd-order bending mode (σ = 0.32). The mode hybridization can also be inferred from the displacement pattern of mode 5 in α-QA shown in Figure 3b: the displacement of the peripheral atoms is clearly reduced compared to a pure rotation (cf., Figure S5), which is consistent with an admixture of the molecular 2nd-order OP bending mode from Figure 2d. The minor contributions of other molecular rotations (around the normal axis with σ = 0.129 and around the long axis with σ = 0.118) reflect the fact that the rotation does not occur exactly around the short molecular axis. The reason for the hybridization of the two modes despite their rather different frequencies is the close molecular packing in α-QA: the above-described interlocking of molecules in neighboring QA layers (see Figure 1c) impedes a simple, rigid rotation of the QA molecules around their short molecular axes. Two other examples of modes arising from a hybridization of inter- and intramolecular vibrations are modes 7 and 9. Both modes are a superposition of the long-axis rotation and again the 2nd-order OP bending. While for mode 7, the rotation component dominates (σ = 0.89 vs 0.46 for the 2nd order bending), the opposite holds true for mode 9. There, a stronger 2^nd^-order bending (σ = 0.82) and a weaker long-axis rotation (σ = 0.43) are combined with a non-negligible short-axis rotation (σ = 0.36). The latter again indicates that the rotational axis is not exactly aligned along one of the molecular axes (which is best seen in the animation of the mode in the Supporting Material). Mode 7 is visualized in Figure 3c,d. In contrast to what would be expected for a rotation around the long molecular axis, certain atoms like the nitrogens and their neighbors are hardly displaced. A similar observation is made for mode 9, albeit here the oxygens and their neighbors remain essentially stationary (see Figure 3e,f). This behavior is attributed to the peculiar situation of the N- and O-atoms caused by the formation of the H-bridges. The above hybridizations illustrate, how intermolecular bonding as well as the crystalline packing cause a modification of the vibrational displacement patterns, which is best described as an intermode hybridization on the basis of the molecular eigenmodes.

### 4.2 Overview of the Phonon Band Structure of α-QA

**Figure 4:**
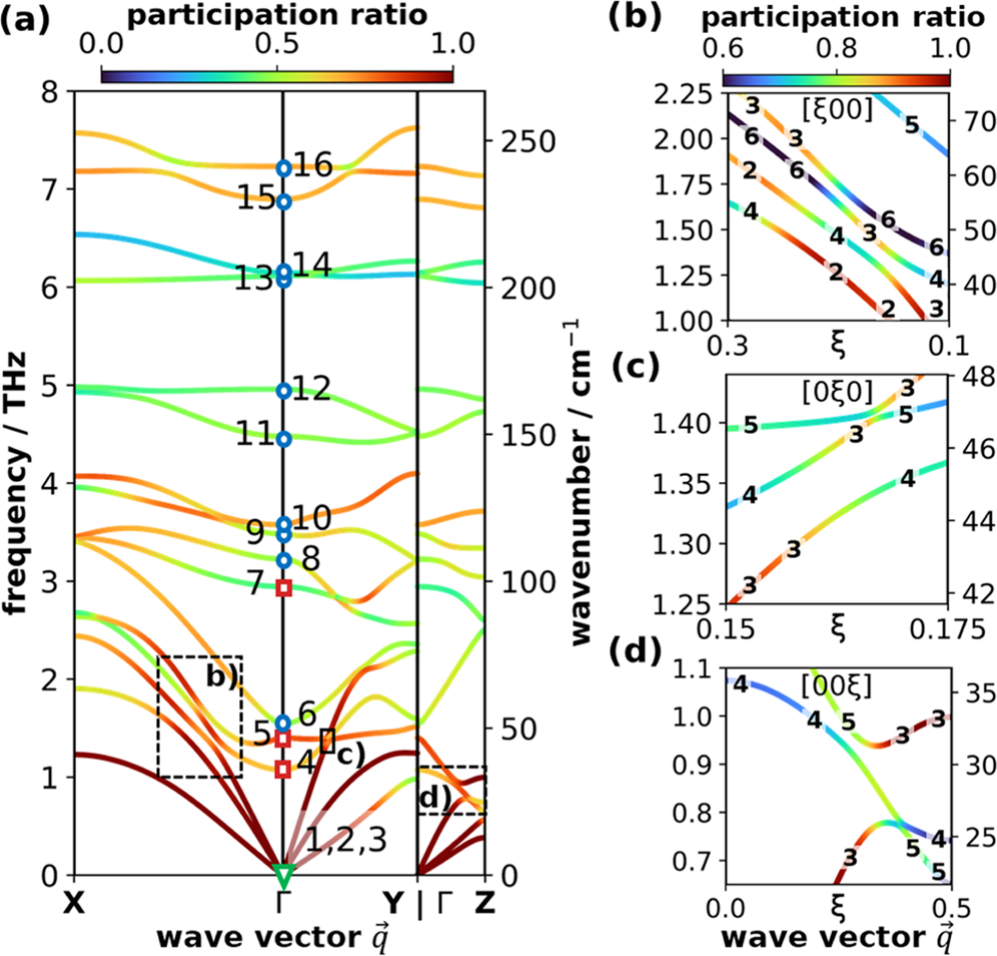
(a) Phonon band structure of α-QA along the most important high-symmetry directions in the 1st Brillouin zone (see Figure 1f) showing the relation between vibrational frequencies (ordinate) and wave vectors (abscissa). Frequencies are presented in units of THz (left axis) and in wavenumbers (right axis) in panels (a)–(d). Phonon bands are colored according to the mode participation ratio (eq 2). For the sake of visibility, a different color code was chosen for panel (a) than for the other panels. Particularly, relevant sections along each band structure path are presented as close-ups in panels (b)–(d). The locations of the close-ups in reciprocal space are indicated by black rectangles in panel (a). The wave vectors of the highlighted sections are shown in reduced coordinates ξ of the reciprocal lattice vectors. Numbers in panels (b)–(d) denote the nature of the displacements characterizing a specific band. The labeling follows the order of specific modes at the Γ-point (cf., Figures 2a and 3a). This illustrates the evolution of modes characterized by specific patterns of atomic motions in the region of avoided crossings between the bands.

In solids containing a quasi-infinite number of atoms, Γ-point vibrations reveal only a small part of the vibrational properties. Therefore, it is useful to analyze phonon band structures. They display vibrational frequencies as a function of the wave vector *q⃗*, which quantifies the phase shift between the displacements in neighboring unit cells. The bands are then usually plotted along high-symmetry directions in reciprocal space. Figure 4a shows such a phonon band structure for α-QA in the low-frequency region with phonon modes colored according to their participation ratios. As discussed in Section 3, these quantify how coherently the atoms move for each vibrational eigenmode and their analysis eases the identification of similar displacement patterns along the bands. The Γ-vibrations discussed in the previous section (cf., Figures 2a and 3a) are labeled by their numbers and by blue circles (internal modes), red squares (rotational modes), and a green triangle (marking the acoustic modes). In α-QA with only one molecule in the unit cell, the latter are the only translational modes. They are of particular relevance, as they are responsible for sound propagation, they often dominate thermal conductivities, and their slopes are intimately related to the material’s elastic constants.^68,69^ Especially, at low *q⃗*-values, the acoustic modes display a high participation ratio owing to their translational character (i.e., all molecules move to the same degree and in-phase in neighboring unit cells).

At larger *q⃗*-values, the bands experience a number of avoided crossings that occur when bands approach, hybridize, and eventually “repel” each other. This happens when the eigenvectors associated with the two bands transform equally under the symmetry operations of the crystal. Due to the low symmetry of the α-QA crystal (space group 1̅), this applies to all pairs of bands, which rules out any band crossings and, thus, maximizes the number of avoided crossings in α-QA. Notably, after the avoided crossing, the character of the mode is often transferred from one band to the other. This is, for example, illustrated by the evolution of the participation ratios in Figure 4a (and more clearly in the zooms in Figure 4b–d), where high participation ratios in the course of an avoided crossing often switch from a lower to a higher band. Therefore, following the *q⃗*-dependent evolution of modes with high participation ratios often yields rather smooth trends, even though individual bands often have a (more or less) pronounced kink close to avoided crossing, as shown, e.g., in Figure 4d, with the character of the lowest mode transitioning from band 3 to band 5.

The first optical band, which is associated with an intermolecular mode, has a Γ-point frequency of 1.07 THz (mode 4 in Figure 4a). It primarily corresponds to a rotation around the axis normal to the molecular plane. Together with the next optical band (mode 5 in Figure 4a), it participates in various avoided crossings with the acoustic bands, as can be seen in more detail in Figure 4b,c. Also, higher-lying bands participate in avoided crossings, as will be discussed in more detail below. Most of the higher bands display a reduced albeit still significant dispersion. All of these observations are strongly reminiscent of the situation in the more commonly studied pentacene crystals.^67^ They consist of molecules of a rather similar size and shape as QA. The 1st Brillouin zone of pentacene, however, contains twice as many bands due to two molecules in the unit cell. A closer inspection of the acoustic bands reveals another distinct difference: in pentacene, the slopes of the longitudinal acoustic band(s) in the Γ*X* and Γ*Y* directions are very similar;^67^ conversely, in α-QA, the band in the Γ*Y* direction is much steeper, testifying to a much higher phonon group velocity of the longitudinal acoustic band in that direction. This can be explained by the different structures of the two materials: in pentacene, due to the herringbone arrangement of the molecules, bonding in the *X*Γ*Y* plane is rather isotropic and mostly of van der Waals type (with quadrupole contributions). Conversely, in α-QA, the Γ*Y* direction is close to the direction of the short molecular axis of the QA molecule (see Figure 1e,f) and thus also close to the direction of the H-bonding interaction. In the spirit of a coupled harmonic oscillator model, a high dispersion in this direction (and, thus, a high group velocity) can be associated with a high coupling force constant. This is fully consistent with the notion that the resistance to longitudinal deformations is particularly high in α-QA due to the reinforcing hydrogen bonds.

The above discussion requiring the (repeated) mentioning of similarities of directions already suggests that the conventional approach of analyzing phonon band structures along high-symmetry directions in reciprocal space might not be ideal for α-QA. In fact, as illustrated in Figure 1e,f, in α-QA, these directions neither coincide with the real-space lattice vectors of the crystal (and, thus, with the 3D arrangement of the molecules) nor with the most relevant geometric directions within the molecules. The latter comprise the short and long molecular axes and the normal to the π-plane, where it should be stressed again that unique identification of these directions is possible only because of the simple structure of α-QA with only a single molecule in the unit cell. In fact, as will be shown below, only in the “molecular” directions, purely longitudinal and transverse acoustic phonons are found and, in these directions, also the maximum group velocities occur. This makes the three “molecular” axes ideally suited for the further analysis of acoustic and optical phonons and suggests that the bands should be plotted also along these directions.

### 4.3 Acoustic Bands of α-QA

**Figure 5:**
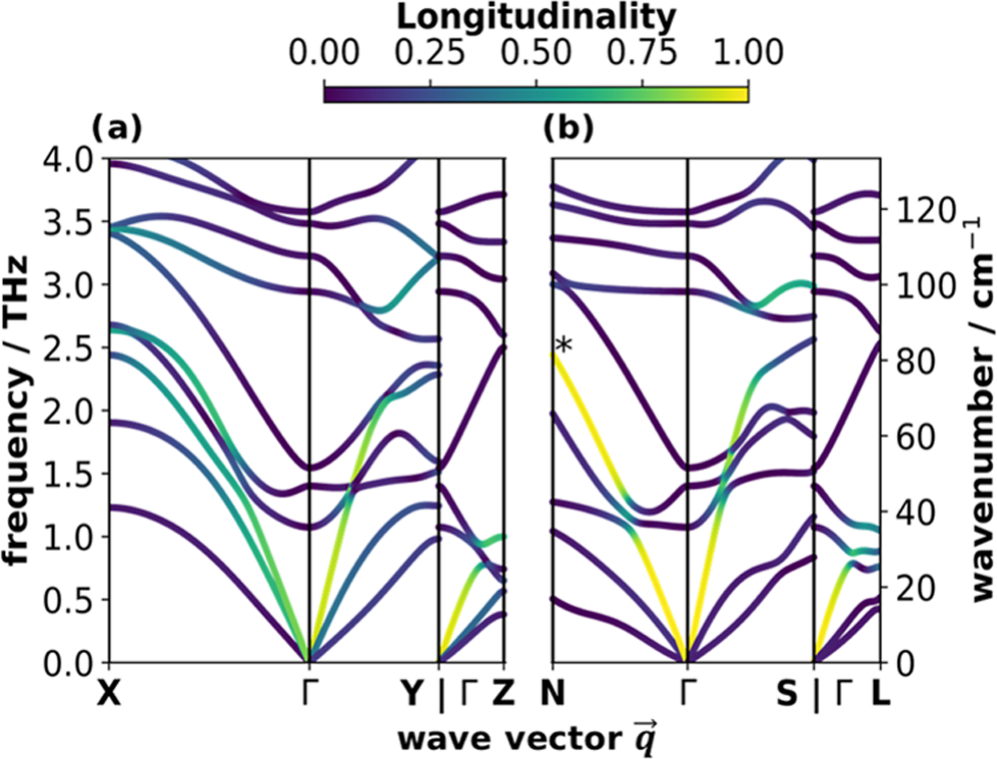
Low-frequency phonon band structures of a-QA colored according to the longitudinality of the modes, which ranges between 0 and 1 (see the color bar at the top). Phonon frequencies are plotted for wave vectors along (a) high-symmetry paths of the 1st Brillouin zone and (b) along paths parallel to the molecular axes (see 1st Brillouin zone in Figure 1f). The asterisk in (b) indicates the longitudinal acoustic mode at N.

Such a plot is contained in Figure 5b, where we now color the bands according to their degree of longitudinality (see Section 3; eq 3). As a complementary graph, Figure 5a illustrates the longitudinality of the modes in the conventional, high-symmetry directions. In the following discussion, we will first focus on the acoustic bands at low *q⃗* values, while the impact of the observed avoided crossings will be discussed later.

The longitudinality of the bands serves as a measure of how parallel atomic displacements are to the wave vector for each mode. Notably, Figure 5a shows that along the high-symmetry directions, a unique identification of a single longitudinal acoustic band is not possible. Especially along the Γ*X* path, two acoustic bands with similar but only intermediate degrees of longitudinality exist. A mixed band character is also found along the Γ*Y* and Γ*Z* paths; although in these two directions, the longitudinal character dominates for the highest-energy acoustic band (at least at low *q⃗* values). Still, also the longitudinality of the second band is not negligible. In fact, a closer analysis of the atomic motions derived from the eigenmodes of the highest acoustic band reveals that it is dominated by a rigid displacement of the QA molecule parallel to the short molecular axis for both the Γ*X* and Γ*Y* directions (see Figure S16b in the Supporting Information).

As argued already at the end of the previous section, a much clearer situation should emerge when analyzing vibrations with *q⃗*-vectors parallel to one of the molecular axes. This is indeed the case, as one can see in Figure 5b: now the highest band in all three directions has a purely longitudinal character. For example, the longitudinality in the ΓN direction (i.e., perpendicular to the planes of the QA molecules) is 0.99 close to the Γ-point and after two avoided crossings still amounts to 0.96 at *N* (for the band highlighted with a “*”). Notably, as mentioned previously for the participation ratio, also the longitudinality switches between bands at the avoided crossings with low-lying optical bands. At small *q⃗*-vectors, similarly high degrees of longitudinality are observed for the third acoustic band also along the ΓS and ΓL paths (i.e., for *q⃗* parallel to the short and long molecular axes). For these directions, the longitudinality of the respective bands, however, decreases toward the Brillouin zone boundary, where the phonon modes hybridize after partaking in avoided crossings with relatively large gaps (see SI Figure S16e,f). It is worthwhile mentioning that the purely longitudinal character of the highest acoustic band in ΓN, ΓS, and ΓL directions means that for the corresponding eigenmodes, the molecules rigidly move perpendicular to the π-plane, parallel to the short molecular axis, and parallel to the long molecular axis, respectively. This now allows a less ambiguous explanation for why bands are particularly steep in certain directions: for example, the longitudinal acoustic band is steepest along the ΓS path (slope of 57 THzÅ = 5.7 nm/ps). The associated displacement along the short molecular axis correlates well with the direction of the reinforcing H-bonds, which stiffens the corresponding vibrations and thus results in particularly high phonon group velocities (see also below). As another example, in the ΓL and ΓS directions, the lowest band with the smallest dispersion is the transverse acoustic band associated with a displacement of the molecules parallel to the plane normal (see Figure S16). This suggests that the smallest restoring force constant is found for a displacement of the molecules parallel to the π-stacking direction, where one is mostly dealing with a combination of van der Waals and electrostatic attraction (due to charge penetration effects)^46^ and Pauli repulsion.^47^ Consistently, also the longitudinal acoustic band in the ΓN direction (which is described by the same displacement) displays the lowest dispersion (amounting to 32 THzÅ = 3.2 nm/ps).

**Figure 6:**
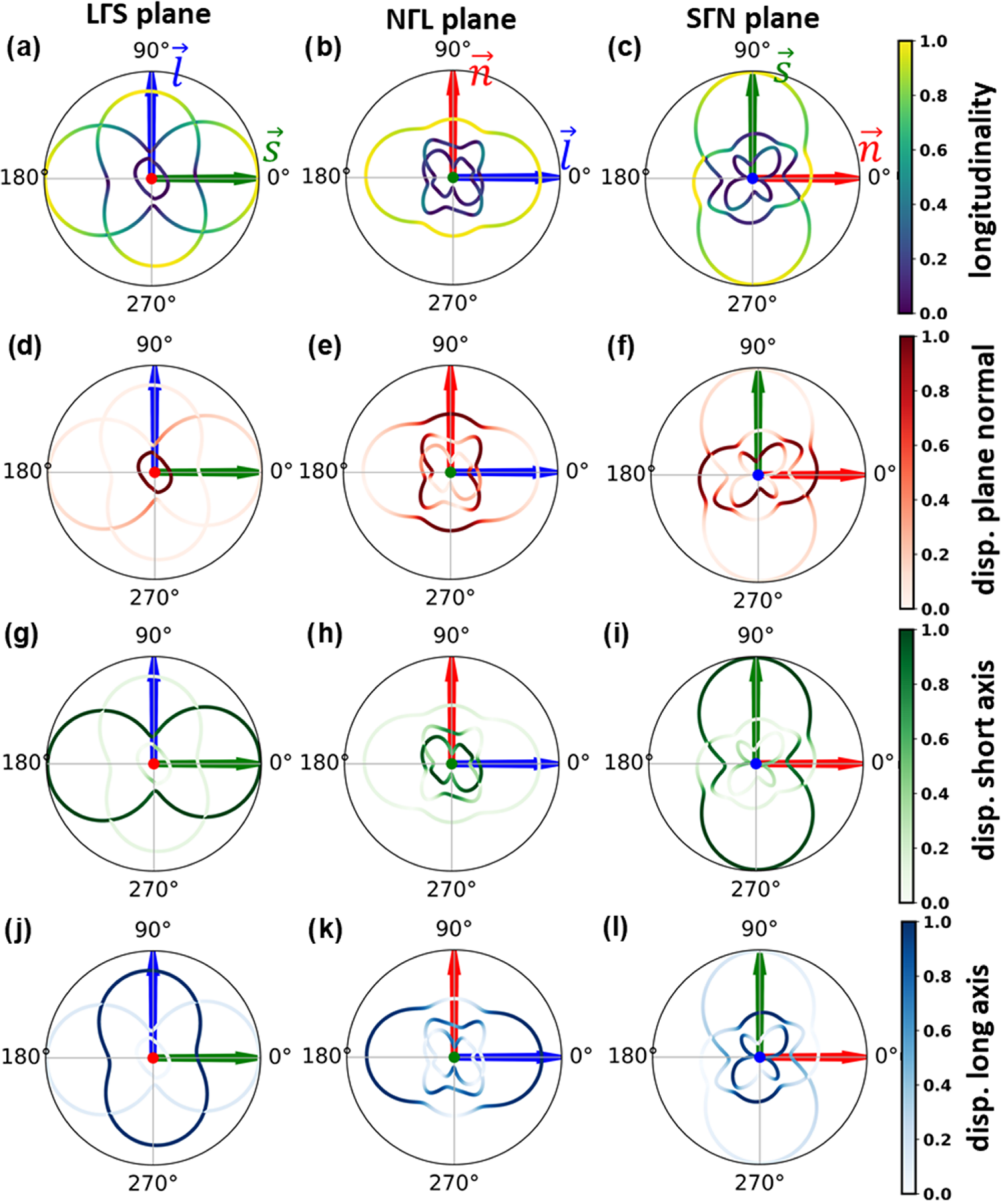
Angular-dependent band structures of α-QA in the long-wavelength limit (for *|q⃗|* = 0.0044 Å ^– 1^, which is 1% of the shortest reciprocal lattice vector) for three circular band paths. The radial values cover a frequency range between 0 and 0.04 THz (1.33 cm^–1^; all plots use the same frequency scale). The frequencies of the three acoustic modes are plotted for wave vectors parallel to the molecular plane (panels a, d, g, j; left column) in the plane spanned by the long molecular axis and the normal to the π-plane (panels b, e, h, k; central column) and in the plane spanned by the short molecular axis and the normal to the π-plane (panels c, f, i, l; right column). The arrows denote the direction of the normal to the π-plane (red arrow), the direction of the short molecular axis (green), and the direction of the long molecular axis (blue). These directions are labeled for each angular band structure in panels (a)–(c). The coloring in the top row indicates the longitudinality of the modes (panels a, b, c), while in the following rows, it quantifies the degree to which the displacements associated with specific modes are parallel to the normal to the molecular plane (panels d, e, f), to the short molecular axis (panels g, h, i), and to the long molecular axis (panels j, k, l), respectively (see also color codes at the right end of each row).

The fundamentally different character of the longitudinal acoustic bands in the *n⃗*, *s⃗*, and *l⃗* directions raises the question whether there is a gradual angle-dependent change of the molecular displacement directions within a single band or whether the order of the bands with constant character changes at certain angles. To understand that, it is useful to study the band structure as a function of the direction of *q⃗* while keeping the length of the wave vector, |*q⃗*|, fixed. Such angular-dependent band structures are rarely explored. Here, they are plotted close to the Γ-point in Figure 6 for a |*q⃗*|-value of 0.0044 Å^–1^, which is 1% of the shortest reciprocal lattice vector. The plots show the phonon frequencies of the first three acoustic bands on a scale between 0 and 0.04 THz along circles in reciprocal space. These circles comprise angular-dependent bands for *q⃗*-vectors within the molecular plane (i.e., in the LΓS plane containing the long and short molecular axis; left column of Figure 6), *q⃗*-vectors in the NΓL plane (i.e., in the plane spanned by the long molecular axis and the normal to the π-plane; central column of Figure 6), and *q⃗*-vectors in the SΓN plane (i.e., in the plane spanned by the short molecular axis and the normal to the π-plane; right column of Figure 6). The plotted frequencies are identical in each column of Figure 6, but their color-coding varies: In the first row, the longitudinality of the modes is shown, while the following rows quantify the degree to which the modes correspond to displacements along the normal to the π-plane (red color code), along the short molecular axis (green color code), and along the long molecular axis (blue color code).

The angular band structures reveal several characteristics of the acoustic phonon bands: (i) there is a pronounced anisotropy of all bands with (local) maxima of the phonon energies occurring along the *l⃗*, *s⃗*, and *n⃗* directions, especially for the outermost (highest frequency) acoustic band. The global frequency maximum occurs in the *s⃗* direction, consistent with the previous discussion of maximum band dispersions. For the lower bands, local maxima also occur in the vicinity of avoided crossings. (ii) The outermost band is always the one with the highest degree of longitudinality. This is the consequence of avoided crossings (including switches in band character) occurring at angles, at which a specific type of mode (long-axis, short-axis, or normal displacement) is no longer the most longitudinal one. (iii) Longitudinalities close to one are observed typically only rather close to the *l⃗*, *s⃗*, and *n⃗* directions. (iv) In these directions, the longitudinality of the lower-frequency modes is essentially zero (i.e., they possess a purely transverse character), while away from the special directions, bands with mixed character are found. (v) The frequency splitting at avoided crossings varies significantly. This results in pronounced differences in the evolutions of the apparent nature of the different bands in different planes of reciprocal space.

Especially for the directions plotted in the second and third columns of Figure 6, it appears that avoided crossings due to hybridization effects prevent bands from maintaining a specific character, in analogy to the situation depicted in Figures 4 and 5. For the azimuthal band structure plotted in the first column of Figure 6 (i.e., when *q⃗* lies in the molecular plane), the situation is somewhat different, especially for the higher frequency bands. For them, the gaps at the avoided crossings are marginal. This means that for the corresponding *q⃗* directions, there is hardly any hybridization between long- and short-axis displacements. Due to the marginal frequency gaps, one might, in fact, get the impression that one is dealing with crossing bands that maintain their character: two dumbbell-shaped outer bands characterized by long- and short-axis displacements and a third inner band for which the molecules are displaced perpendicular to the π-plane. The two outer bands appear to intersect at an angle of 52°, where the longitudinality of both bands adopts a value of 0.707 (=√2/2). However, for symmetry reasons, there must be avoided crossings also for these bands even though they are hardly visible in Figure 6. This is confirmed by a zoom into the corresponding *q⃗*-region that is shown in the Supporting Information (Figure S18). Therefore, strictly speaking, one is dealing with a continuous highest frequency band, whose character abruptly changes between long- and short-axis displacement at the positions of the avoided crossings. Similarly, also for the band structures plotted in the NΓL and SΓN planes, the character of the highest energy band switches between either normal, long-, or short-axis displacement depending on the wave-propagation direction but in a much more gradual fashion. Concomitantly, there are larger frequency splitting at the avoided crossings.

### 4.4 Optical Bands in α-QA and Their Involvement in Avoided Crossings

As the next step, it is worthwhile to provide a more in-depth discussion of the optical bands. They are mostly characterized by participation ratios noticeably smaller than those of the acoustic modes. This can be understood from the fact that they correspond to vibrations for which significant parts of the molecules move comparably little. Somewhat higher participation ratios in Figure 4a are found only for short- and normal axis rotations and for the in-plane bending mode (modes 4, 5, and 10 at the Γ-point). As mentioned above, the participation ratio is a useful guide for following specific mode characters throughout the bands in cases in which strong mode hybridizations occur in the vicinity of avoided crossings. At these points also the participation ratio changes rather abruptly, which is a strong indication for a fundamentally changed nature of the bands in question. As an example, band 4, which at Γ corresponds to a rotation around the plane normal, partakes in two avoided crossings approximately halfway toward *X*, as shown in Figure 4a,b. There, it hybridizes with the two acoustic bands 2 and 3. At small values of |*q⃗*|, band 4 is comparably flat, while in response to the avoided crossing bands 4 and 5 at higher |*q⃗*|-values adopt rather sizable dispersions. Rather, at large values of |*q⃗*|, close to *X*, band 2 becomes comparably flat (Figure 4a). This can be attributed to its increasingly optical character (as can be inferred from the low associated participation ratio, an assessment that is confirmed by an inspection of the eigenmodes). Similar effects occur at many points in reciprocal space, where strongly dispersing bands with acoustic displacement characteristics approach an optical band and undergo an avoided crossing at which the band characters switch. Notably, such avoided crossings of optical and acoustic phonons are typically detrimental to heat transport.^70−72^ The situation can become rather complex, as can be illustrated for the mode dominated by a rotation around the short molecular axis. Close to Γ, such vibrations form band 5 (with a Γ-point frequency of 1.40 THz). At the *X*-point in Figure 4a, after some avoided crossings including switching of band characters, the corresponding vibration appears as mode 8 at 3.45 THz (as shown in more detail in Figure S17). Conversely, along Γ*Y* (see Figure 4c) and Γ*Z* (see Figure 4d), the negative dispersion and avoided crossings result in this vibration appearing at 0.98 THz at *Y* (as band 1) and at 0.65 THz at *Z* (as band 3).

Notably, avoided crossings also occur between purely optical bands, which shall be exemplified for bands 7, 8, and 9 along Γ*Z*: as discussed above, when comparing molecular vibrations with Γ-point phonons in the crystal, at the Γ-point, bands 7 (Figure 3c,d) and 9 (Figure 3e,f) are closely related and consist of superpositions (Figure 3a) of a long-axis rotation and a 2nd-order OP bending motion. Conversely, band 8 corresponds to the first-order torsion. Along the Γ*Z* path, the bands switch their character multiple times such that the long-axis rotation, which dominates band 7 at the Γ-point, becomes band 8 at *Z*, while the 2nd order OP bending, which is dominant for band 9 at Γ, becomes band 7 at *Z* (see Figure S17b). Concomitantly, the first-order torsion switches from band 8 at Γ to band 9 at *Z*. Interestingly, as a consequence of rehybridizations at avoided crossings, the original Γ-point hybridizations of the eigenmodes for bands 7 and 9 disappear at *Z*, such that there the displacements can be directly correlated with individual molecular eigenmodes of QA. Similar decouplings of the long-axis rotation and the 2nd order OP bending modes are also observed at *X* and *Y*.

### 4.5 Group Velocities

Phonon-related transport characteristics are, to a large extent, determined by the group velocities, *v⃗*_*ν*,*q⃗*_, of the phonons with the indices referring to the band number, *ν*, and the wave vector, *q⃗*. This becomes apparent, for example, for the thermal conductivity tensor κ^αβ^, which (employing the Boltzmann transport equation in the relaxation-time approximation) is given by^73,74^
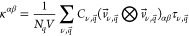
6

Here, *V* is the unit cell volume and *N*_*q*_ refers to the number of considered *q⃗*-points in the sampling of reciprocal space, while the *C*_ν,*q⃗*_ are the mode-specific heat capacities and the τ_*ν*,*q⃗*_ are the respective phonon lifetimes. The mode-specific heat capacities are derived from the phonon energies and from the phonon occupations in thermodynamic equilibrium and are given by^73^
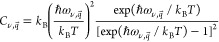
7with the mode eigenfrequencies ω_ν,*q⃗*_ and the temperature *T*. Considering that the group velocities are the local slopes of the phonon band structures, their values for the different bands can be estimated already from the band plots in the band structures in Figures 4 and 5 and from the absolute values of the phonon frequencies in the angular-dependent phonon bands in Figure 6. For their more quantitative assessment, we pursued two approaches: first, group velocities of the acoustic modes were calculated in the long-wavelength limit for small wave vectors close to the Γ point in analogy to the angular-dependent phonon band structures in Figure 6. Second, group velocities were calculated for all vibrational modes on a dense *q⃗*-mesh sampling the entire 1st Brillouin zone. Then, they were weighted according to their temperature-dependent occupation based on their *C*_ν,*q⃗*_-values to assess their potential contribution to quantities like thermal conductivity.

**Figure 7:**
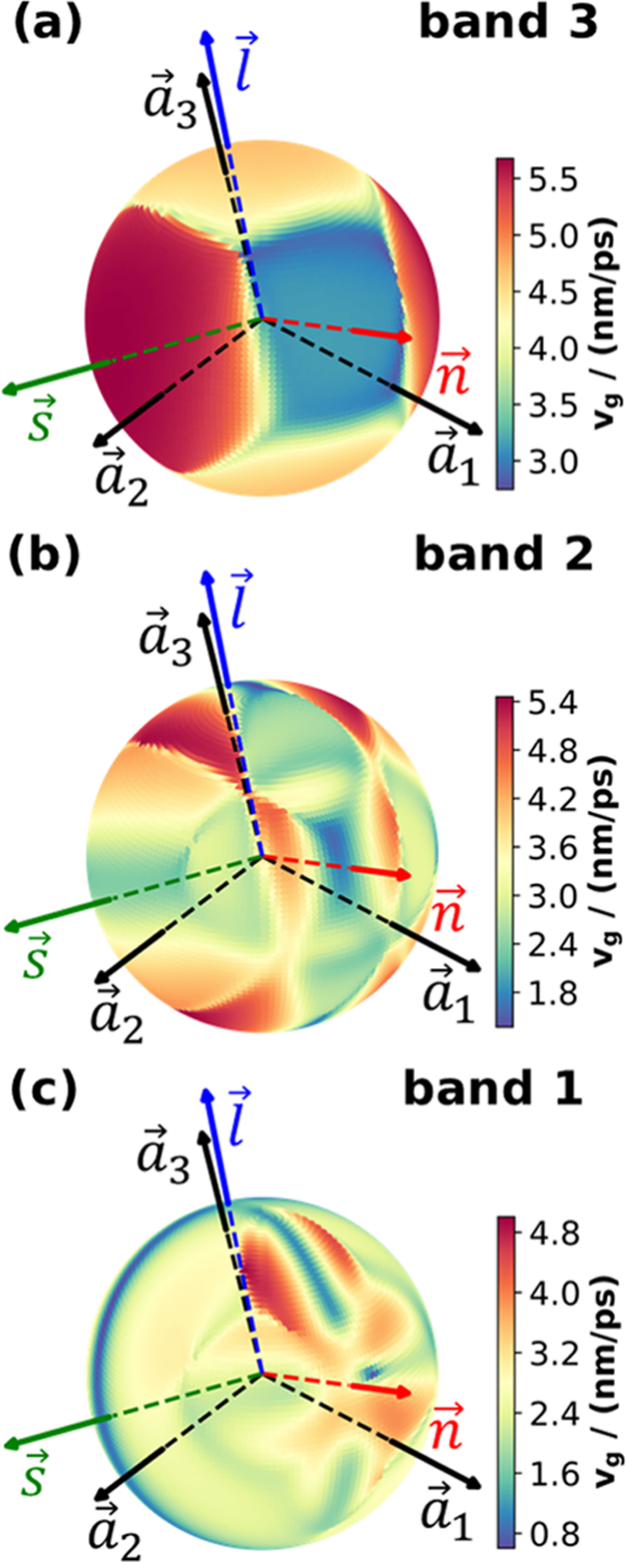
Angular dependence of the norms of the group velocities |*v⃗*_*g*_*|* denoted as *v*_g_ for the three acoustic phonon bands in the long-wavelength limit (for |*q⃗*| = 0.0044 Å^–1^). Panel (a) shows the situation for the highest frequency, mostly longitudinal acoustic band, while panels (b) and (c) show the situations for bands 2 and 1, respectively. The lattice vectors *a⃗*_1_, *a⃗*_2_, and *a⃗*_3_ are denoted by the black arrows, while the red, green, and blue arrows denoted the direction of the molecular plane normal *n⃗*, the short *s⃗*, and the long axis *l⃗*. Note that to better visualize the angular dependences, the total ranges of *v*_g_ vary between the different plots as indicated in the color scales to the right.

In the long-wavelength limit, (i.e., close to Γ), the acoustic bands exhibit perfectly linear dispersion and their slopes with respect to *q⃗* do not change with |*q⃗*|. They rather depend on the direction of *q⃗*, as can be inferred from the section on angular-dependent band structures. To illustrate that, the norms of the group velocities |*v⃗*_g_| are plotted for the three acoustic bands (again at |*q⃗*| = 0.0044 Å^–1^) and in all spatial directions in Figure 7.

The structures of these plots are rather complex. The reason for that are the avoided crossings discussed already in the context of the angle-dependent band structures. They result in abrupt changes of |*v⃗*| as a function of the *q⃗* direction, resulting in nearby regions with group velocities significantly above and significantly below the average values. For band 3 (the mostly longitudinal band), for which |*v⃗*| is plotted in Figure 7a, one can still see certain trends: the highest group velocities with 5.7 nm/ps (5.7 km/s) are found for phonon propagation directions parallel to the short molecular axis (green arrow in Figure 7a); another local maximum is found along the long molecular axis (blue arrow). Between these directions, one observes a kink in the evolution of |*v⃗*| consistent with the above-discussed very “pointy” avoided crossing of the two highest frequency bands in the left column of Figure 6. The particularly high group velocity along the short molecular axis can again be attributed to the particularly strong interactions in the H-bonding direction. Conversely, in the π-stacking direction (red arrow in Figure 7a), one observes a very low group velocity. A more in-depth analysis reveals that the minimum group velocities are found approximately halfway between that direction and the direction parallel to the long molecular axis (i.e., approximately half way between the red and blue arrows). As shown already in Figure 6, for the mostly transverse bands (bands 1 and 2), the repeated avoided crossings result in distortions of the angular-dependent band structures with sometimes multiple local frequency minima and maxima and particularly pronounced local frequency variations. This then translates into the extremely complex variations of the group velocities in the polar plots of Figure 7b,c for which an identification of clear trends appears futile.

As the final step, we analyze the group velocities of the optical phonons together with the acoustic phonons in the entire 1st Brillouin zone. As a detailed discussion of all modes would explode the contents of the current manuscript, we opted for “collectively” analyzing their contributions to the thermal conductivity. According to eq 6, the key phonon properties that determine thermal transport are the mode-specific heat capacities, the phonon group velocities, and the phonon lifetimes. The latter are inherently anharmonic quantities and their determination requires the knowledge of third- and possibly even higher-order force constants.^16,73^ As strictly anharmonic quantities, they are out of the scope of the present manuscript (and clearly beyond what is currently computationally accessible for ab initio methods applied to systems as complex as α-QA). Rather, we will consider the mode-resolved contributions to the thermal conductivity that depend purely on harmonic properties. They are given by the dyadic product of the group velocities weighted by the respective mode contributions to the heat capacity (with the latter accounting for thermal occupation of the phonon modes). This yields what we refer to as the harmonic contributions to the thermal anisotropy tensor, η^αβ^ (in analogy to what was done in ref (15))
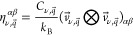
8

To homogeneously sample reciprocal space in the calculation of η_*ν*,*q⃗*_^αβ^, phonon frequencies and group velocities were calculated on a 56 × 34 × 15 *q⃗*-mesh. Summing over the tensors for all modes weighted by the phonon lifetimes gives the thermal conductivity tensor, while merely summing over the η_*ν*,*q⃗*_^αβ^ yields a proportional quantity, if all phonon lifetimes were the same. While the latter is usually not the case, η^αβ^ still provides a first impression of which phonons have harmonic properties that would make them relevant for thermal transport. Moreover, our preliminary tests suggest that at least in α-QA, there is no pronounced anisotropy of the phonon lifetimes, such that the anisotropy of η^αβ^ yields at least a first hint toward the anisotropy of the thermal conductivity.

**Figure 8:**
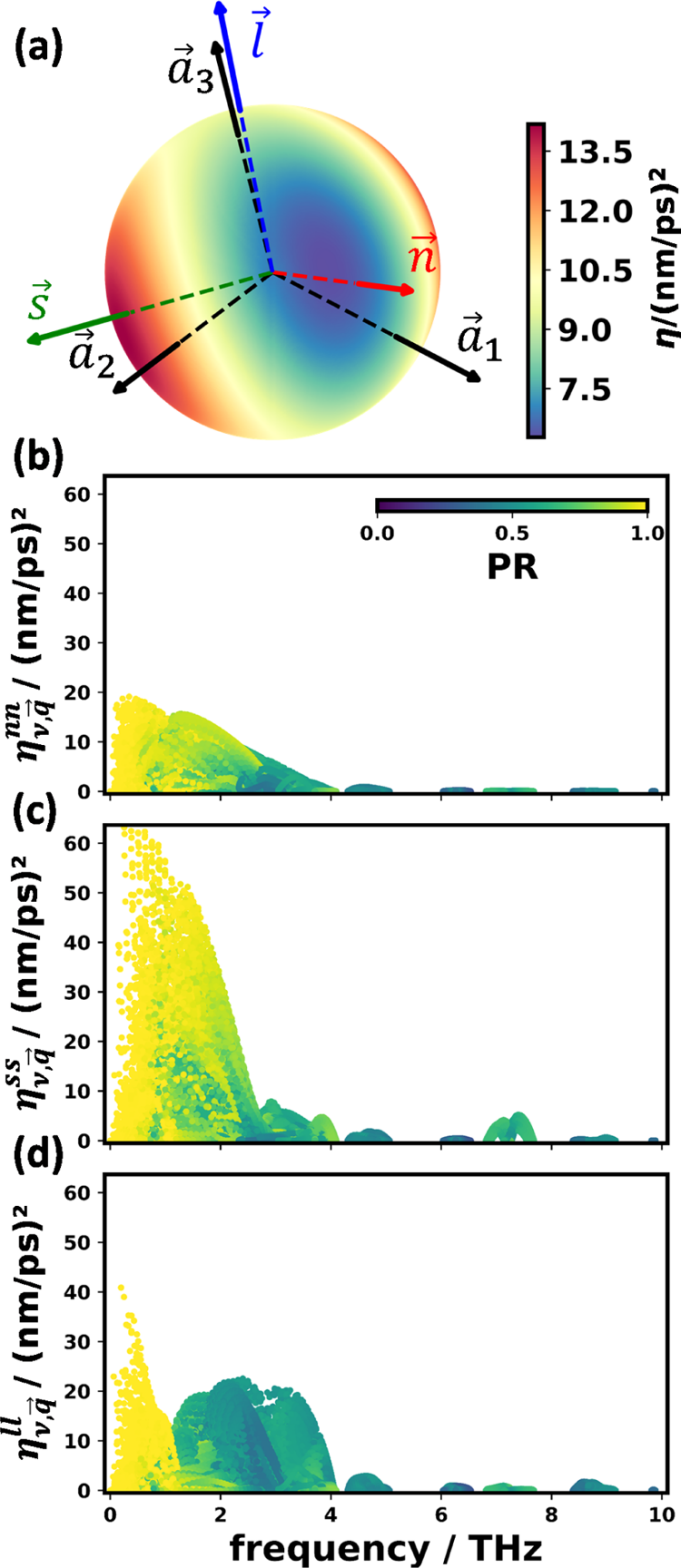
(a) Polar plot of the harmonic contributions to the thermal anisotropy tensor summed over all modes projected onto unit vectors. As a consequence of the latter, the displayed quantity relates a temperature gradient in a specific direction to the component of the heat flux in that direction. The lattice vectors *a⃗*_1_, *a⃗*_2_, and *a⃗*_3_ are denoted by the black arrows, while the red, green, and blue arrows denote the direction of the molecular plane normal *n⃗*, the short *s⃗*, and the long axis *l⃗*. (b–d) Harmonic contributions to the thermal anisotropy tensor η^αβ^ in directions perpendicular to the molecular plane, η^*nn*^ (b); parallel to the short molecular axis, η^*ss*^ (c); and parallel to the long molecular axis, η^*ll*^ (d). The data points are colored according to the respective mode participation ratios.

Therefore, a polar plot of a projection of η^αβ^ (the sum over all mode contributions described in eq 8) calculated for a phonon occupation found at a temperature of 300 K is shown in Figure 8a. The projections are obtained by multiplying the tensor from both sides with a unit vector pointing in a specific direction. Thus, the plotted quantity describes the contribution to the component of the heat flux in the given direction for a temperature gradient in the same direction. The angular dependence of the projection of η^αβ^ is much smoother than that of the group velocities of the individual bands in Figure 7. We primarily attribute that to the averaging over several bands and, even more importantly, over the entire Brillouin zone. It, however, also has to be mentioned that when sampling reciprocal space on the surface of a sphere (like for Figure 7), a significantly denser *q⃗*-grid can be chosen than for a three-dimensional (3D) sampling of the entire 1st Brillouin zone (like for Figure 8). Nevertheless, also when considering all bands and the entire 1st Brillouin zone, several of the original results from the analysis of the acoustic group velocities at small |*q⃗*| are recovered, like a particularly high value of η^αβ^ in the direction of the short molecular axis, *s⃗*, an intermediate value along the long molecular axis, *l⃗*, and the smallest value perpendicular to the molecular plane, *n⃗*.

To understand that in more detail, the contributions of individual phonons in these directions were also analyzed. For that, the mode thermal anisotropy tensors were transformed to align their axes with *s⃗*, *l⃗*, and *n⃗* (η_*ν*,*q⃗*_^*xy*^≔ *x⃗*^*T*^·η⃡·*y⃗* for *x*, *y* = *n*, *s*, *l*). The diagonal elements of the mode contribution tensors are shown in Figure 8b for η_*ν*,*q⃗*_^*nn*^, in panel (c) for η_*ν*,*q⃗*_^*ss*^, and in panel (d) for η_*ν*,*q⃗*_^*ll*^. They provide additional insight, why phonon transport should be particularly efficient in the H-bonding direction (with 63 nm^2^/ps^2^ as the highest contribution of η_*ν*,*q⃗*_^*ss*^), intermediate parallel to the long molecular axis (40 nm^2^/ps^2^ as the highest contribution of η_*ν*,*q⃗*_^*ll*^), and smallest in the π-stacking direction (20 nm^2^/ps^2^ as the highest contribution of η_*ν*,*q⃗*_^*nn*^).

Figure 8b–d shows that the contributions of the acoustic phonons (more specifically, of all phonons with high participation ratios) to  are particularly large in the direction of the short molecular axis. This becomes especially apparent in panel (c) of Figure 8, where particularly high values of η^*ss*^ are found at low frequencies for modes displaying a bright yellow shading (denoting a high participation ratio of the respective modes). The contributions of the related bands are clearly reduced in the directions perpendicular to the molecular plane (Figure 8d) and parallel to the long molecular axis (Figure 8b). Notably, in the latter direction, there are rather sizable contributions from modes with low participation ratios between 1 and 4 THz. This is consistent with the data in Figure 4, where the bands in that spectral region are characterized by rather low participation ratios in the Γ*Z* direction (which is close to *l⃗*), which once more illustrates that, when combining the different elements discussed in this manuscript, a consistent picture emerges.

## 5 Summary and Conclusions

In summary, due to its comparably simple structure, the α-polymorph of QA is ideally suited for studying the intricacies of phonon band structures of highly anisotropic organic semiconductors. As a useful starting point for such an analysis, we identify, how the eigenmodes of the molecules translate into Γ-point vibrations in the molecular crystals. Interestingly, intermolecular rotational modes occur in the same frequency range as the lowest-energy intramolecular backbone-bending vibrations. The order of the intramolecular modes that are found for the isolated molecule is largely maintained in the molecular crystal, although one generally observes a shift to higher frequencies in the latter case as a consequence of intermolecular interactions. These interactions, especially in the form of a geometric interlocking of neighboring molecules, can also be identified as driving forces for mode hybridization effects in α-QA already for Γ-point phonons.

The situation becomes significantly more complex when considering the phonon bands in the entire 1st Brillouin zone. There, the low symmetry of α-QA causes a multitude of avoided crossings. They are often accompanied by abrupt changes of the character of the individual bands, which can be directly inferred from analyzing mode participation ratios and mode longitudinalities. Avoided crossings not only arise from hybridizations between acoustic and optical modes but also repeatedly occur between (sometimes multiple) optical modes. An analysis of the character of the acoustic modes reveals that bands with unambiguous longitudinal or transverse character do not occur in the high-symmetry directions of reciprocal space. In fact, they are found only for phonon wave vectors parallel to the long and short molecular axes and the plane normal of individual QA molecules. The reason for that is that acoustic phonons are characterized by displacements that are parallel to these “molecular” directions. This means that, at least in α-QA, the crystalline packing has only rather little impact on the displacement directions of the molecules when exciting acoustic phonons.

Thus, our observation that the highest frequency acoustic mode always displays the highest degree of longitudinality implies that the nature of that mode must change as a function of the direction of *q⃗*. Analyzing angular-dependent band structures (i.e., phonon frequencies as a function of the direction of *q⃗* rather than of its length) shows that this change in the nature of the involved phonons occurs abruptly again as a consequence of avoided band crossings (now upon changing the direction of the phonon wave vector).

Due to the multiple avoided band crossings, the angular dependence of the group velocities of the acoustic bands (in the long-wavelength limit) adopts a particularly complex structure. One observes massive local changes of the group velocities with kinks and/or local minima and maxima upon only slightly varying the direction of *q⃗*. Still, there are certain trends that prevail throughout all studied quantities: (mostly) longitudinal modes are associated with the largest band dispersions and, consequently, with the largest group velocities independent of the wave-propagation direction and the associated dominant type of molecular displacement. This suggests that longitudinal deformations (i.e., compressions and expansions) are consistently energetically more costly than transverse deformations (i.e., slips of neighboring molecules), despite the highly anisotropic structure of α-QA with well-defined π-stacking and H-bonding directions. Still, the bonding anisotropy has a profound impact on the absolute magnitude of the band dispersions and group velocities (even when averaging over all thermally occupied modes) with the highest values found for deformations in the H-bonding direction and the smallest values occurring for displacements perpendicular to the molecular planes. This is consistent with the notion that bonding interactions are reinforced by H-bonds, while perpendicular to the π-plane, the combination between van der Waals attraction, charge penetration, and (typically rather strong) exchange repulsion results in a much more shallow bonding potential.

Overall, the considerations in the present manuscript not only portray the power of the toolbox available for analyzing phonon band structures in complex materials. They also show that despite a large number of phonon bands in complex materials and the multitude of avoided crossings resulting from the often-low symmetries of organic semiconductors, atomistic simulations allow gaining an in-depth understanding of the properties of phonons as key quasi-particles of crystalline materials. In many cases, these phonon properties can eventually even be traced back to a more intuitive understanding of bonding in molecular crystals. Still, at this stage, the development of dependable structure-to-property relationships for the phonon properties of organic semiconductors is in its infancy, but we are confident that the current study forms a firm basis for future investigations.

## Data Availability

The data underlying this study will be made openly available at the time of publication of the article in the NOMAD repository at https://doi.org/10.17172/NOMAD/2023.05.19-5. The data is also openly available on the repository of Graz University of Technology at https://doi.org/10.3217/zznj9-hd255.

## Supporting Information Available

Animations of the low-frequency Γ-point vibrations of α-QA. A document describing the following aspects: unit cell parameters of α-QA; details on the used basis sets and other electronic structure code settings; a comparison of experimental and calculated hydrogen bonding distances in several QA polymorphs, for PBE and the hybrid functional PBE0; convergence tests for the k-grid and the basis set; details on calculating vibrations of isolated molecules; visualization of the molecular eigenmodes; comparison of the frequencies and participation ratios for all molecular and Γ-point vibrations and their eigenvector overlap; test of the impact of the employed van der Waals correction; tests concerning supercell convergence, where the phonon band structure is presented for additional high-symmetry paths; a variety of tests elucidating the role of anharmonicities; an analysis of displacement types of acoustic phonons along both the high-symmetry paths and the molecular axes; additional details on the avoided crossings in both the high symmetry and the angular band structures (PDF)
Gamma_mode_animations (ZIP)
