# Supplementary material for: Anisotropic Phonon Bands in H-Bonded Molecular Crystals: The Instructive Case of α-Quinacridone
Source: ACS Mater Au. 2023 May 26;3(4):371–85. doi: 10.1021/acsmaterialsau.3c00011 (PMC10347688; doi:10.1021/acsmaterialsau.3c00011)
Supplement: Supplementary file 1 — mg3c00011_si_001.pdf [file mg3c00011_si_001.pdf]

Supporting Information

Anisotropic phonon bands in H-bonded molecular crystals: the instructive case of α-quinacridone

Lukas Legenstein, Lukas Reicht, Tomas Kamencek, Egbert Zojer*

Institute of Solid State Physics, Graz University of Technology, Petersgasse 16, 8010 Graz, Austria

*Email: egbert.zojer@tugraz.at

The data underlying this study will be made openly available at the time of publication of the article in the NOMAD repository at [LINK WILL BE GENERATED, WHEN PAPER HAS BEEN ACCEPTED] The data is also openly available on the repository of Graz University of Technology at https://doi.org/10.3217/zznj9-hd255

1. Structure of α-quinacridone:

The structure investigated of α-quinacridone investigated in this study is based on the structure available in the Cambridge Crystallographic Data Centre (CCDC). Lattice parameters of the experimental unit cell and of the cell optimized with FHI-aims[1] are shown in Table S1.

Table S1: Experimental lattice parameters of quinacridone's α polymorph taken from the CCDC (Identifier: QNACRD06, deposition number: 620257) and lattice parameter optimized with FHI-aims.

	experimental	optimized
a_1 / Å	3.8017	3.845
a_2 / Å	6.6115	6.433
a_3 / Å	14.485	14.645
α / °	100.68	101.09
ß / °	94.40	96.36
γ / °	102.11	100.99

2. Methodological aspects

<u>Details on the used basis sets:</u>

The basis functions employed in the FHI-aims simulations have the format

$$\Phi(r) = \frac{u(r)}{r} * Y_{lm}(\Theta, \Phi)$$

in spherical coordinates (r, Θ, Φ) relative to a given atomic center. FHI-aims provides for every atomic species a preconstructed *species_defaults* file. The used basis sets were not further adjusted (seeTable S2), because they afforded the required accuracy and efficiency. The "intermediate" and "tight" basis sets mentioned in the following section imply the use of basis functions listed in Table S2. Basis set "intermediate" includes all functions up to the first function of "second tier" and basis set "tight" includes all functions listed.

Table S2: Basis functions that have been used for all calculations performed with FHI-aims[1]. The abbreviations read as follows[*]: X(nl, z), where X describes the type of basis function where H stands for hydrogen-like functions and ionic for a free-ion like radial function. The parameter n stands for the main/radial quantum number, l denotes the angular momentum quantum number (s, p, d, f, …), and z denotes an effective nuclear charge, which scales the radial function in the defining Coulomb potential for the hydrogen-like function. In the case of free-ion like radial functions, z specifies the onset radius of the confining potential. If auto is specified instead of a numerical value, the default onset is used.

	H	C	N	O
Minimal	valence (1s, 1.0)	valence (2s, 2.0)	valence (2s, 2.0)	valence (2s, 2.0)
		Valence (2p, 2.0)	valence (2p, 3.0)	valence (2p, 4.0)
First tier	H(2s, 2.1)	H(2p, 1.7)	H(2p, 1.8)	H(2p, 1.8)
	H(2p, 3.5)	H(3d, 6)	H(3d, 6.8)	H(3d, 7.6)
		H(2s, 4.9)	H(3s, 5.8)	H(3s, 6.4)
Second tier	H(1s, 0.85)	H(4f, 9.8)	H(4f, 10.8)	H(4f, 11.6)
	H(2p, 3.7)	H(3p, 5.2)	H(3p, 5.8)	H(3p, 6.2)
	H(2s, 1.2)	H(3s, 4.3)	H (1s, 0.8)	H (3d, 5.6)
	H(3d, 7.0)	H(5g, 14.4)	H(5g, 16)	H(5g, 17.6)

[*] As described in the FHI-aims manual, version January 23, 2017

	H(3d, 6.2)	H(3d, 4.9)	H(1s, 0.75)

Further numerical settings, such as the potential cut-offs and details on the integration grid were the same for both basis sets and remained unchanged from the default settings of “tight” as described in the FHI-aims manual (FHI-aims: A User’s Guide: March 4, 2021).

Details on the Self-Consistent-Field cycle and other simulation settings:

Settings for the calculations presented in the main manuscript include the “relativistic atomic_zora scalar” tag, the spin set to “none”, and specific accuracies for the convergence of the self-consistency (SC) cycle in FHI-aims. These criteria were based on 1.) charge density (sc_accuracy_rho), 2.) total energy (sc_accuracy_etot), 3.) sum of eigenvalues (sc_accuracy_eev), and 4.) forces (sc_accuracy_forces) and their (very tight) values are listed in Table S3 below.

Table S3: Self-Consistency cycle convergence criteria accuracies for the charge density (rho), the total energy (etot), the sum of eigenvalues (eev), and the forces.

sc_accuracy_rho	5e-07
sc_accuracy_etot	1e-08
sc_accuracy_eev	5e-05
sc_accuracy_forces	1e-05

DFT-relaxed hydrogen bonding distances:

To verify that the H-bonding (O···H-N) in α-QA is correctly computed with dispersion-corrected density-functional theory and more specifically the PBE functional, we compared computed intermolecular O—N distances with those of the experimental structures from Paulus et al. (ref. 3). This is the only structure solution of the α-polymorph of QA on the CCDC and it was based on powder diffraction, involving a Rietveld refinement. This typically results in increased uncertainties of the precise atomic positions compared to single-crystal diffraction data. Therefore, we extend the comparison to the structures of the β- and γ-polymorphs, which were solved from single-crystal X-ray diffraction data and show different molecular stackings.

The atomic positions within the experimental unit cells where relaxed with FHI-aims to a maximum residual force component of 1e-03 eV/Å. The computational settings involve the use of PBE[3], a Generalized Gradient Approximation (GGA) functional, with either “tight” or “intermediate” basis-sets and PBE0[4], a hybrid functional, with “intermediate” basis-sets. The k-grid dimensions for the

“intermediate” calculations were also reduced to 4×3×2 from 6×4×2. The non-local many-body-dispersion correction (MBD-NL)[5] was applied in each calculation. This vdW correction was shown to drastically improve the description of molecular complexes of the S66 set[6] and molecular crystals of the X23 set[7] (both containing hydrogen bonded materials) compared to either PBE or PBE0 alone. The comparison of H-bonding distances is summarized in Table S4. , which shows the O—N distance and in brackets the difference between experiment and computation in units of Å for the computational settings discussed above. For the polymorphs whose structure we regard as more reliable, the differences between experiment and calculation are -0.018 Å and -0.042 Å, which we regard as excellent agreement, with both showing a slight over-binding. The experimental O—N distance of the α-polymorph is lower than for the similarly stacked β-polymorph (0.135 Å) alluding to the uncertainty in the structure solution of α and neither PBE or PBE0 relaxation exactly closely reproduces the distance of 2.750 Å. Still the calculated values are rather close to the experiment. The difference between the hybrid and the GGA functional is rather low (0.031 Å), however when we consider the over-binding seen for PBE in β- and γ-QA, PBE0’s higher intermolecular O—N distance should be in slightly better agreement (which could only be proven with more reliable single-crystal data for α-QA). PBE0 comes with the drawback of increased computational cost, with one SCF cycle taking roughly 18 times longer than with PBE for the “intermediate” basis calculations with reduced k-grid and 132 times longer for the tight settings. These findings suggest, that the combination of PBE and the non-local many-body-dispersion reasonably reproduces the intermolecular O···H-N bonding geometry and is applicable to study these systems and that, albeit slight improvements can be gained with the hybrid functional PBE0, the increased in computational costs does not warrant its use.

Table S4: Intermolecular O—N distances of experimental atomic positions from Paulus et al.[3] *and the DFT-relaxed atomic positions and their difference in units of Å.*

	polymorph		
	α	β	γ
Paulus et al.	2.750 (+0)	2.885 (+0)	2.797 (+0)
PBE, tight basis	2.855 (+0.105)	2.867 (-0.018)	2.755 (-0.042)
PBE, inter. basis	2.849 (+0.099)	-	-
PBE0, inter. basis	2.881 (+0.131)	-	-

For the above comparison, the experimental unit cell was used, as the experiments had been performed at room temperature. This is, however, only of minor relevance, as for the seomewhat smaller, fully PBE+MBD-NL optimized unit cell, the O-N distance decreases only marginally by 0.037 Å.

3. Convergence of the k-grid and the basis set

The k-point sampling influences the total energy of unit cell obtained in the calculations. Based on the convergence test shown in Figure S1, we chose the Γ-centered 6×4×2 k-mesh for all calculations of the primitive unit cell of α-QA. When studying the 4×3×2 super cell, the scaled and further expanded 2×2×1 grid was used.

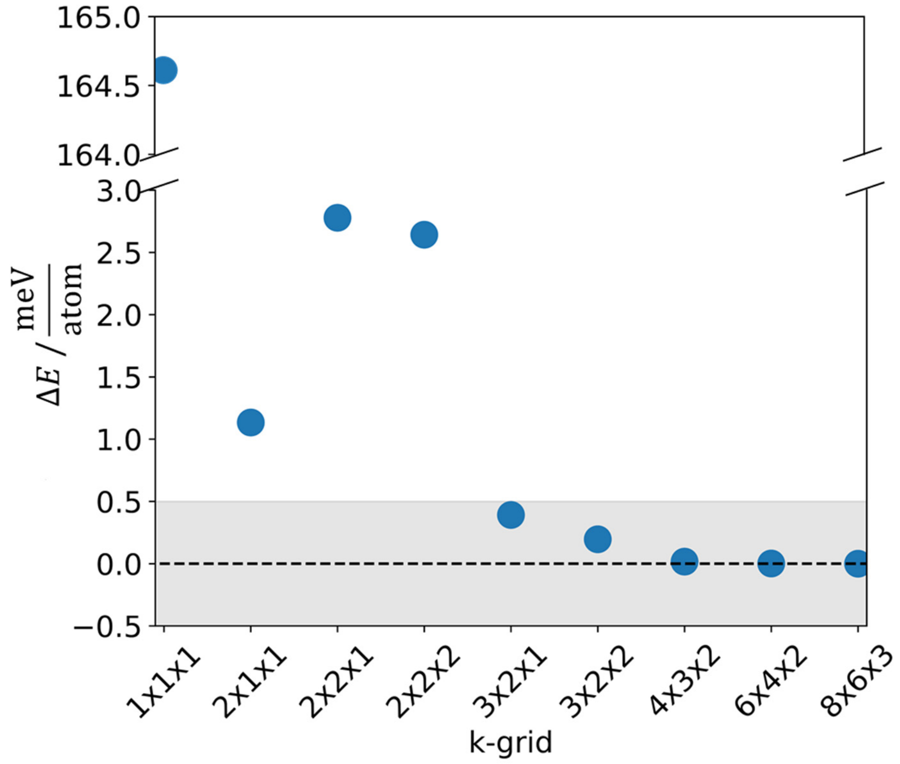

Figure S1: Energy per atom of α-QA as a function of the size of the k-grid. The energies are given relative to the value obtained with the 8×6×3 grid.

To assess the impact of the k-point mesh on the vibrational frequencies, Figure S2, compares the Γ-point vibrations calculated with the 6×4×2 mesh to those obtained with the over-converged 8×6×3 mesh. Also, the eigenvectors associated with the two k-meshes are essentially identical, which further confirms that a 6×4×2 k-mesh is fully sufficient here.

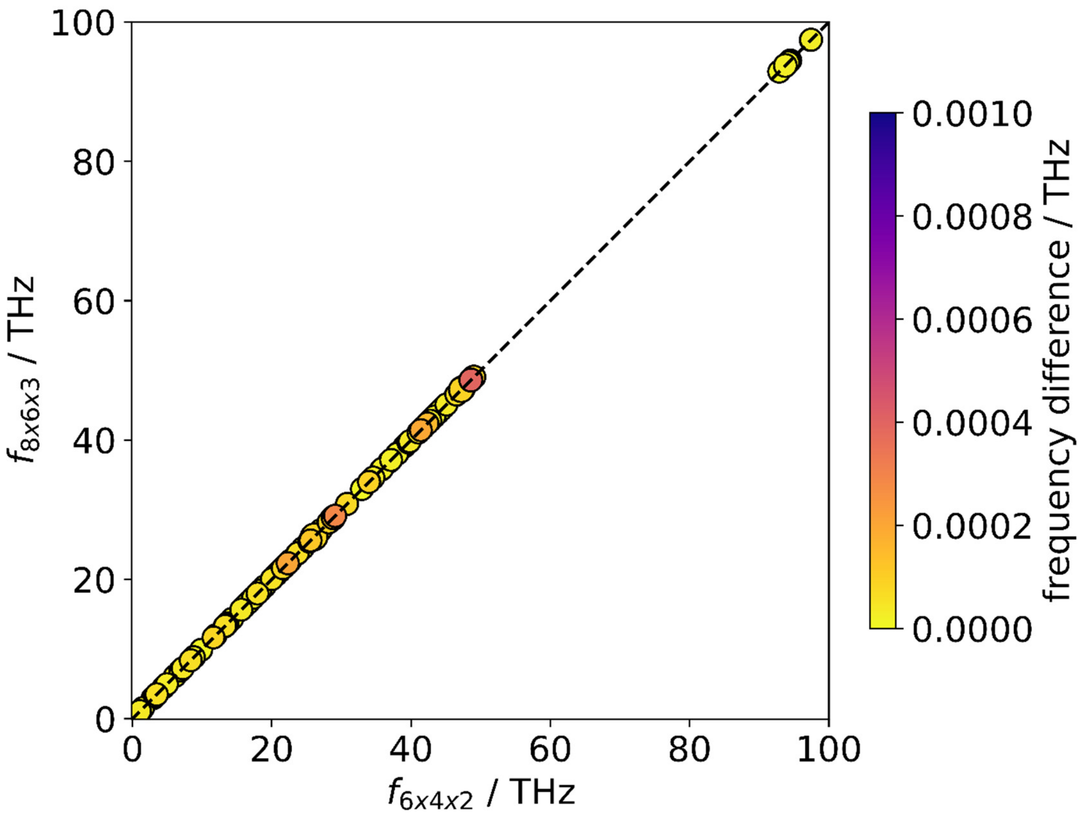

Figure S2: Γ-frequency comparison for 6×4×2 and 8×6×3 k-point meshes. The coloring shows the absolute frequency difference between the two settings.

To test the impact of the used basis set, we calculated the vibrational frequencies for two of the default FHI-aims basis sets, namely "intermediate" and "tight" (see Table S2). For the vast majority of the modes, the differences between the "tight" basis-set and the "intermediate" basis-set are insignificant. Only higher-lying modes show slight deviations both in frequencies and eigenvectors (larger and darker data points in Figure S3). These eigenvector deviations are simply $(1 - \sigma_{mn}) \cdot 100$ in percent, where σ_{mn} refers to the eigenvector overlap as discussed in the main work (Eq. 1), but for two equivalent modes calculated using the two basis sets. They are indicated by the size of the data points. These modes are either localized on the hydrogen or oxygen atoms. Applying an "intermediate" basis would, therefore, be already sufficient for the low-frequency vibrations. Nevertheless, to be on the safe side, we consistently applied a default "tight" basis.

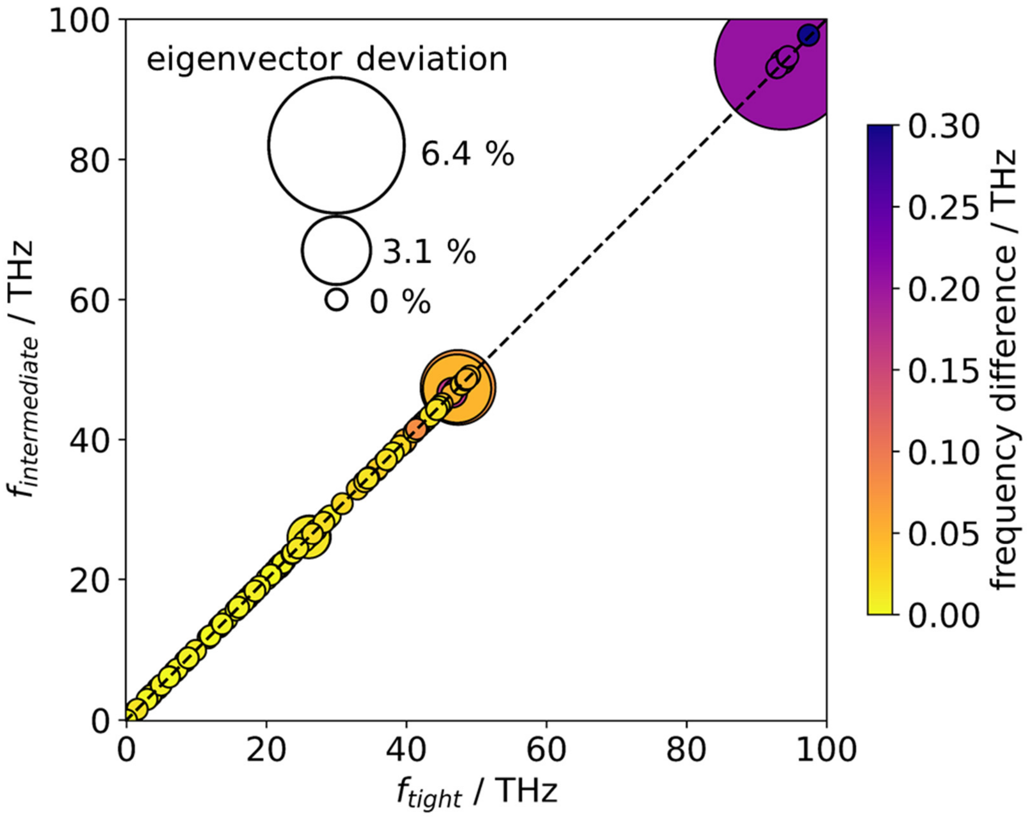

Figure S3: Γ-frequency comparison for the "tight" and "intermediate" default basis sets of FHI-aims. The coloring additionally shows the absolute frequency difference between the two. The size of the data points shows the deviation from a perfect overlap of the eigenvectors for the respective mode calculated with the two basis sets (for details see main text).

4. Calculating vibrations of isolated molecules

In Figure S4, a comparison of vibrational frequencies of molecular QA obtained with different approaches is shown. These approaches comprise FHI-aims[1] calculations employing open and periodic boundary conditions (where for the latter a very large unit cell was used). More details are provided in the method section of the main paper. The results of the periodic and open-boundary FHI-aims calculations are essentially identical, as shown in Figure S4. This is also reflected in a marginal

frequency root-mean-square deviation (RMSD) between the periodic and the open approach of 0.0012 THz (0.04 cm^{-1}).

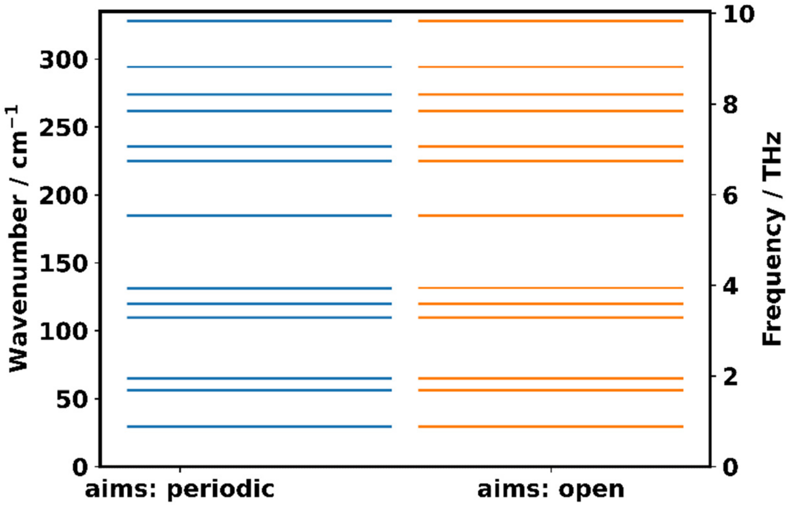

Figure S4: Comparing frequencies of molecular QA in the low-frequency region which is representative of all modes for the two different approaches. blue lines: data for a periodic system with a 100×100×100 Å³ unit cell evaluated with Phonopy; orange lines: molecular system evaluated with get_vibrations.py from the FHI-aims utilities

5. Visualization of molecular eigenmodes of molecular quinacridone

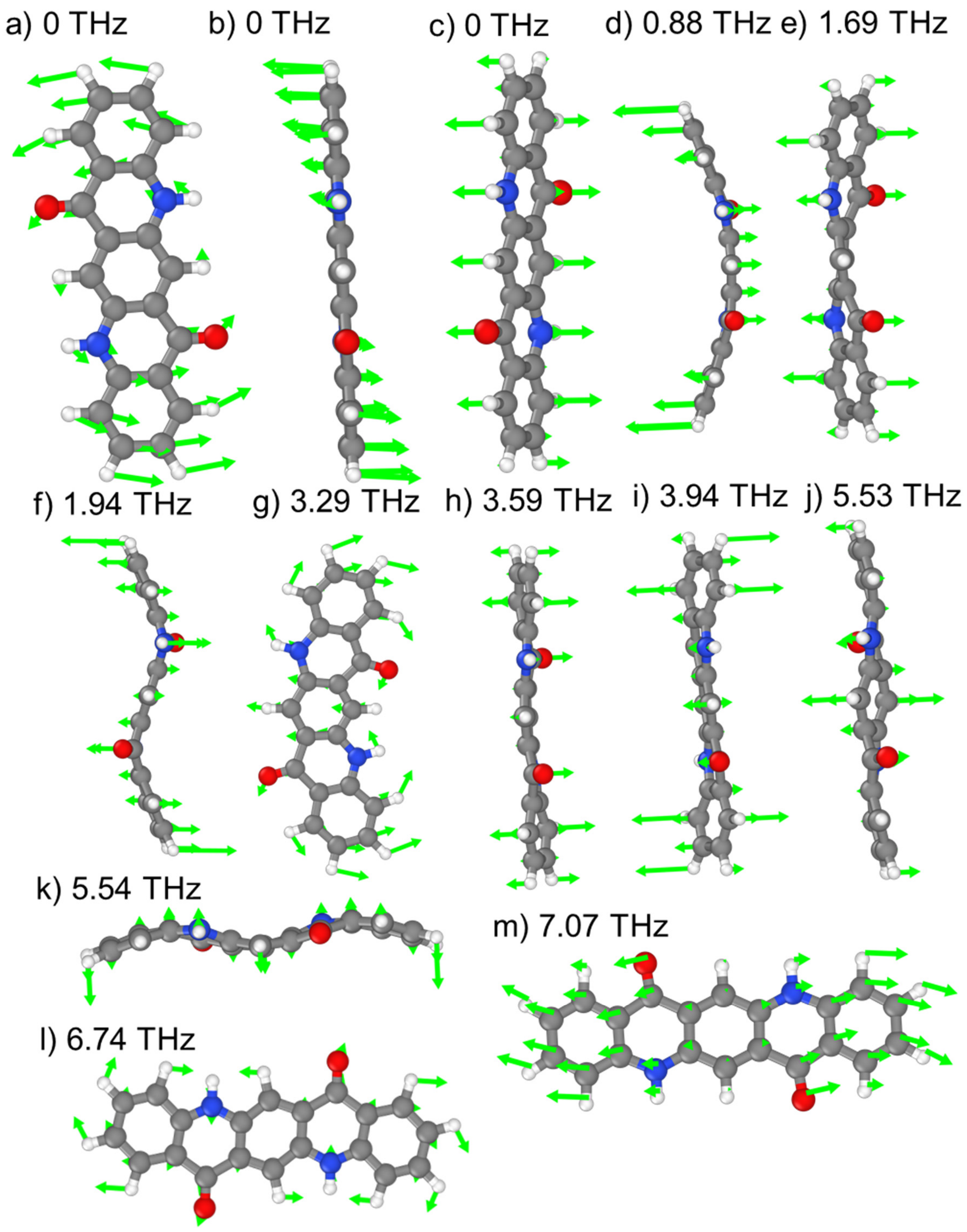

Figure S5: Visualization of the eigenmodes for the low-frequency molecular vibrations of QA. Rotation a) plane normal, b) short axis, and c) long axis. d) 1st-order OP bending, e) 1st-order torsion, f) 2nd-order OP bending, g) 1st-order IP bending, h) torsion rings 1,5, i) 2nd-order torsion, j) torsion ring 3, k) 3rd-order bending, l) 2nd-order IP bending, and m) long axis stretching.

6. Vibrational frequencies: molecular QA compared to the crystalline α-phase

The vibrational frequencies of QA in the gas phase and in the α-phase were matched according to the largest eigenvector overlap σ_{nm}(see Equation 1 and Figure 3a). The frequencies, largest overlap elements and the mode participation ratios are shown in Table S5. The numbers in Table S5 represent the data from Figure S6.

Table S5: Vibrational frequencies of molecular QA, f_{mol}, and Γ-frequencies of α-QA, f_α, in units of terahertz (THz) and wavenumbers (cm^{-1}) for each of the 108 (eigen)modes. Both, the molecular vibrations and Γ-frequencies were calculated with the Phonopy package employing periodic boundary conditions. Therefore, the molecular rotation modes 4, 5, and 6 show very small, but not exactly zero frequencies. Additionally, the largest eigenvector overlap elements for each mode and the mode participation ratios for both the molecular modes and Γ-modes of α–QA are shown.

Mode	f_{mol} / THz	f_α / THz	f_{mol} / cm^{-1}	f_α / cm^{-1}	max(σ_{nm})	PR_{mol}	PR_α
1	0.00	0.00	0.0	0.0	0.73	1	1
2	0.00	0.00	0.0	0.0	0.73	1	1
3	0.00	0.00	0.0	0.0	1	1	1
4	0.12	1.07	4.2	35.8	0.98	0.63	0.65
5	-0.07	1.40	-2.3	46.7	0.93	0.55	0.76
6	-0.16	2.94	-5.3	98.2	0.98	0.43	0.44
7	0.88	1.54	29.4	51.5	0.89	0.55	0.4
8	1.69	3.23	56.4	107.6	0.9	0.49	0.48
9	1.95	3.48	65.0	116.2	0.82	0.47	0.5
10	3.29	3.57	109.6	119.2	0.91	0.66	0.68
11	3.59	4.48	119.8	149.3	0.98	0.45	0.49
12	3.94	4.96	131.5	165.4	0.98	0.4	0.44
13	5.54	6.14	184.6	204.9	0.99	0.46	0.38
14	5.54	6.12	185.0	204.0	0.99	0.29	0.3
15	6.74	6.90	224.9	230.0	0.98	0.67	0.68
16	7.07	7.23	235.7	241.2	0.98	0.62	0.7
17	7.84	8.39	261.7	279.7	0.98	0.32	0.34
18	8.22	8.82	274.1	294.2	0.99	0.45	0.48
19	8.82	8.92	294.2	297.5	0.98	0.49	0.48
20	9.84	9.88	328.2	329.7	0.99	0.35	0.36
21	11.22	11.66	374.1	388.9	0.98	0.52	0.59
22	12.02	11.96	401.0	398.8	0.99	0.33	0.3
23	13.15	13.32	438.5	444.4	0.79	0.2	0.26

**24**	13.20	13.42	440.5	447.8	0.93	0.21	0.22
**25**	13.36	13.24	445.6	441.8	0.8	0.69	0.36
**26**	13.42	13.67	447.7	455.8	0.95	0.56	0.58
**27**	14.09	14.33	470.1	478.0	0.99	0.76	0.75
**28**	15.06	21.80	502.4	727.0	0.95	0.26	0.22
**29**	15.11	22.64	503.9	755.1	0.97	0.3	0.25
**30**	15.79	15.64	526.8	521.6	0.98	0.49	0.55
**31**	15.86	16.02	529.1	534.4	0.99	0.73	0.73
**32**	15.90	16.55	530.4	552.0	1	0.39	0.43
**33**	16.29	16.79	543.3	559.9	0.98	0.31	0.33
**34**	17.27	17.34	576.2	578.4	0.98	0.63	0.6
**35**	17.87	17.97	596.1	599.5	1	0.68	0.68
**36**	18.33	18.35	611.5	612.2	0.97	0.31	0.4
**37**	18.86	19.00	629.0	633.6	0.98	0.48	0.48
**38**	19.97	20.04	666.0	668.6	0.95	0.32	0.4
**39**	20.30	20.62	677.1	687.9	0.97	0.17	0.21
**40**	20.54	20.66	685.0	689.3	0.96	0.23	0.21
**41**	21.08	21.15	703.1	705.5	0.63	0.56	0.12
**42**	21.47	21.60	716.3	720.4	0.72	0.06	0.11
**43**	22.04	21.72	735.0	724.5	0.8	0.19	0.22
**44**	22.29	22.11	743.5	737.6	0.92	0.2	0.21
**45**	22.30	22.32	743.8	744.4	0.64	0.06	0.1
**46**	23.55	23.64	785.7	788.4	0.97	0.23	0.34
**47**	23.57	23.75	786.2	792.1	0.95	0.24	0.31
**48**	24.17	24.47	806.1	816.3	0.92	0.41	0.19
**49**	25.24	25.41	841.9	847.7	0.96	0.17	0.21
**50**	25.25	25.59	842.4	853.6	0.96	0.17	0.21
**51**	25.98	26.04	866.7	868.6	0.75	0.35	0.15
**52**	26.38	26.12	879.8	871.3	0.75	0.06	0.1
**53**	26.45	26.57	882.2	886.2	0.99	0.06	0.07
**54**	26.66	26.14	889.4	871.9	1	0.53	0.58
**55**	26.93	27.08	898.2	903.2	0.98	0.4	0.35
**56**	28.36	28.20	946.1	940.6	0.93	0.15	0.19
**57**	28.37	28.16	946.2	939.3	0.95	0.15	0.2
**58**	28.51	28.69	951.1	956.8	0.92	0.15	0.23
**59**	29.13	29.11	971.8	971.0	0.88	0.16	0.24
**60**	29.13	28.87	971.8	963.1	0.96	0.16	0.2
**61**	30.84	30.83	1028.7	1028.3	1	0.22	0.23
**62**	30.84	30.81	1028.9	1027.7	1	0.22	0.23
**63**	32.82	32.96	1094.8	1099.4	0.99	0.28	0.25
**64**	33.00	33.01	1100.8	1101.1	0.99	0.19	0.22
**65**	33.56	34.01	1119.6	1134.4	0.96	0.2	0.22
**66**	33.59	33.98	1120.6	1133.4	0.99	0.09	0.1
**67**	34.41	34.47	1147.8	1149.9	0.99	0.16	0.18

**68**	34.42	34.60	1148.3	1154.1	0.97	0.17	0.19
**69**	35.67	35.85	1189.8	1196.0	0.99	0.12	0.1
**70**	35.74	35.72	1192.3	1191.6	0.98	0.22	0.19
**71**	36.75	36.89	1226.0	1230.6	0.95	0.2	0.2
**72**	37.00	37.14	1234.3	1238.9	0.99	0.28	0.29
**73**	37.72	38.00	1258.2	1267.5	0.92	0.22	0.25
**74**	37.80	38.09	1260.8	1270.6	0.95	0.17	0.23
**75**	38.44	39.10	1282.4	1304.2	0.9	0.33	0.29
**76**	38.51	39.64	1284.7	1322.3	0.94	0.15	0.25
**77**	39.21	39.82	1308.1	1328.3	0.97	0.39	0.24
**78**	40.39	41.07	1347.1	1370.0	0.95	0.36	0.37
**79**	41.10	41.41	1371.1	1381.3	0.96	0.63	0.7
**80**	41.19	41.76	1373.8	1393.0	0.98	0.45	0.51
**81**	41.89	42.37	1397.2	1413.4	0.91	0.15	0.23
**82**	42.25	42.76	1409.2	1426.5	0.96	0.28	0.27
**83**	43.24	43.34	1442.2	1445.6	0.99	0.21	0.22
**84**	43.59	43.90	1453.9	1464.2	0.93	0.35	0.29
**85**	44.10	44.29	1471.0	1477.4	0.98	0.27	0.3
**86**	44.52	44.68	1485.0	1490.5	0.97	0.38	0.39
**87**	44.70	45.09	1491.1	1504.1	0.97	0.31	0.31
**88**	45.95	46.45	1532.7	1549.2	0.93	0.19	0.33
**89**	46.85	47.36	1562.7	1579.8	0.47	0.67	0.32
**90**	47.13	47.23	1572.0	1575.3	0.81	0.21	0.18
**91**	47.28	47.81	1577.2	1594.7	0.87	0.52	0.67
**92**	47.95	46.52	1599.5	1551.8	0.74	0.18	0.17
**93**	48.13	48.44	1605.5	1615.8	0.89	0.24	0.11
**94**	48.43	48.60	1615.3	1621.2	0.87	0.58	0.34
**95**	48.93	46.84	1632.0	1562.6	0.81	0.35	0.22
**96**	49.50	49.03	1651.3	1635.4	0.79	0.41	0.3
**97**	92.71	92.93	3092.6	3099.8	0.63	0.08	0.07
**98**	92.72	92.89	3092.7	3098.5	0.63	0.08	0.07
**99**	92.97	94.39	3101.2	3148.4	0.75	0.17	0.17
**100**	93.02	94.45	3102.8	3150.6	0.68	0.15	0.1
**101**	93.22	93.73	3109.4	3126.4	0.79	0.15	0.14
**102**	93.22	93.76	3109.6	3127.5	0.74	0.17	0.16
**103**	93.52	93.74	3119.4	3126.8	0.98	0.14	0.12
**104**	93.53	93.73	3119.7	3126.6	0.97	0.14	0.11
**105**	93.82	94.15	3129.6	3140.3	1	0.06	0.06
**106**	93.84	94.20	3130.1	3142.3	1	0.06	0.06
**107**	105.95	97.39	3534.0	3248.7	1	0.06	0.06
**108**	105.96	97.44	3534.4	3250.2	1	0.06	0.06

As there are 108 vibrational modes in both the isolated molecule and the crystalline α-phase, the eigenvector overlap matrix is rather large and quite sparse, hence we present only the overlap elements illustrated in Figure 3a of the main work in Table S6 (however, with the molecular modes ordered by frequency).

Table S6: Eigenvector overlap matrix shown for the lowest twelve vibration modes of isolated QA and α-QA calculated via the scalar product defined in Equation 1.

		**eigenmode number in α-QA**											
	#	1	2	3	4	5	6	7	8	9	10	11	12
**eigenmode number in molecular QA**	1	0.68	0.73	0	0	0	0	0	0	0	0	0	0
	2	0.73	0.68	0	0	0	0	0	0	0	0	0	0
	3	0	0	1	0	0	0	0	0	0	0	0	0
	4	0	0	0	0.98	0.13	0	0.03	0	0.1	0	0	0.05
	5	0	0	0	0.09	0.93	0	0.04	0	0.36	0	0	0
	6	0	0	0	0.08	0.12	0	0.89	0	0.43	0	0	0.07
	7	0	0	0	0	0	0.98	0	0.18	0	0.02	0.01	0
	8	0	0	0	0	0	0.18	0	0.9	0	0.4	0.08	0
	9	0	0	0	0.11	0.32	0	0.46	0	0.82	0	0	0.07
	10	0	0	0	0	0	0.05	0	0.4	0	0.91	0.12	0
	11	0	0	0	0	0	0.02	0	0.02	0	0.14	0.98	0
	12	0	0	0	0.06	0.02	0	0.03	0	0.09	0	0	0.98

Frequencies of both the gas- and Γ-point α-QA modes are plotted in Figure S6 and colored according to their mode participation ratio (Equation 2). Further, all modes of the two phases are connected by black lines, where the transparency was adjusted to the corresponding eigenvector overlap value between 0 and 1. Modes with a perfect overlap are connected by fully intransparent black lines, while for modes with no overlap the lines are fully transparent and therefore invisible. For the α-QA modes 5, 7 and 9 which are explicitly discussed in the main text in section 4.1 and presented in Figures 3b-f,

there are, for example, two or more visible connecting lines, as these modes are superpositions of two or more molecular modes (see Table S6). In the frequency region between 20 and 50 THz (Figure S6) these superpositions are even more common, as emphasized by the connecting lines.

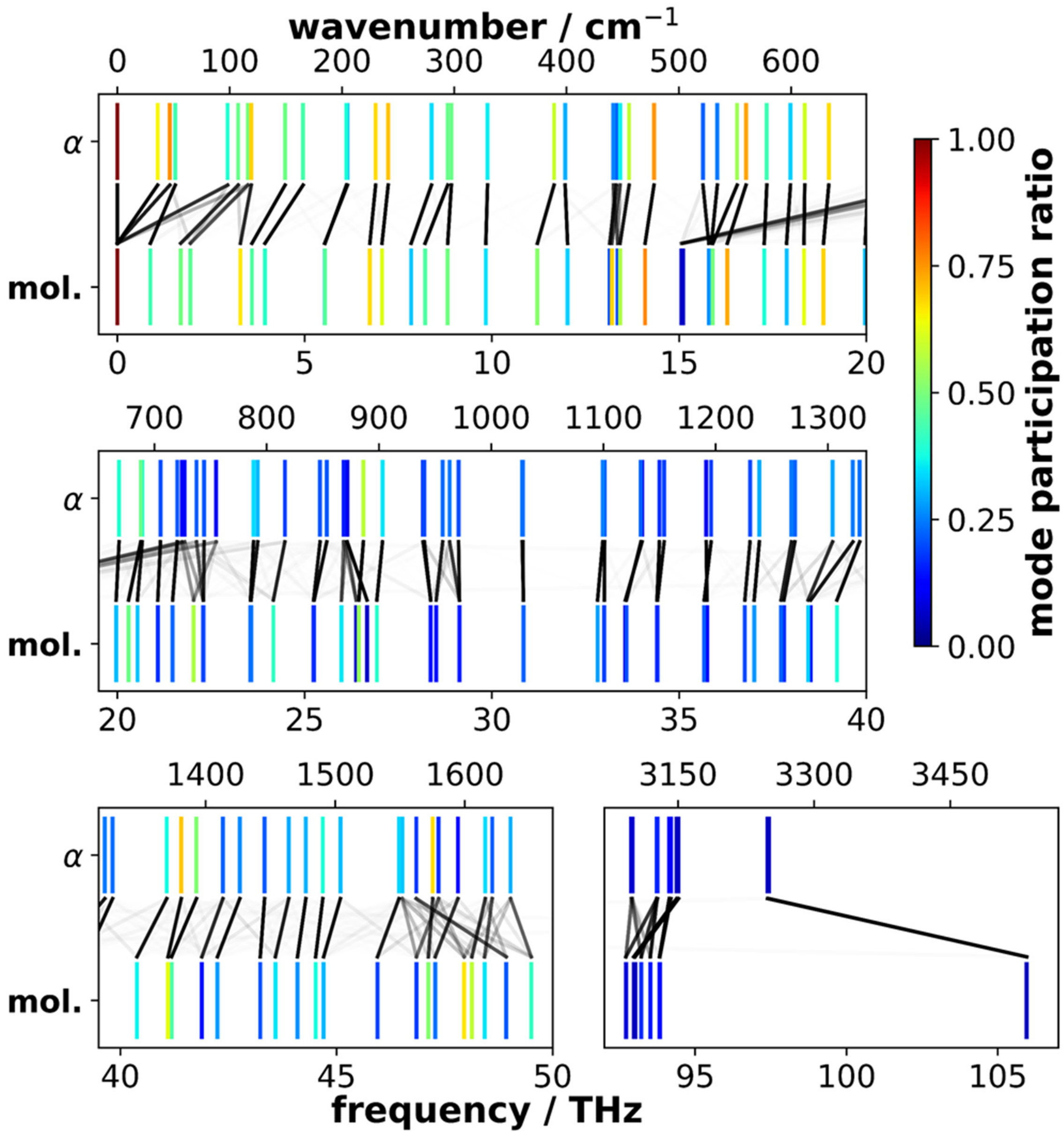

Figure S6: Vibrational modes of the gas-phase and of α-QA (at the Γ-point), with their vibrational frequency presented in units of THz (bottom) and wavenumbers (top). The color indicates the mode participation ratio, with values between 0 (dark blue) and 1 (dark red), as defined in the color bar. The black connecting lines show the eigenvector overlap between the molecular and crystalline vibrations with the transparency of the line scaled by the overlap.

7. Impact of the used van der Waals correction: comparison between FHI-aims and VASP calculations

As mentioned in the introduction of the main manuscript, we have thoroughly benchmarked, which van der Waals corrections most accurately reproduce Γ-point vibrations and phonon bands of organic semiconductor crystals.[8,9] These calculations showed that amongst the tested methods, the best results can be obtained using Grimme's D3(BJ)[10,11] or the many-body dispersion correction (MBD)[12]. All these tests relied on the VASP code with converged numerical settings. In the present calculations, we used FHI-aims in combination with the more efficient non-local many body dispersion (MBD-NL) van der Waals correction by Hermann et al.[5] (which to date to the best of our knowledge is not yet available in VASP). Therefore, in Figure S7 we compare converged VASP phonon band structures using the D3(BJ) correction with FHI-aims calculations discussed throughout the remainder of this manuscript. The VASP calculations were performed with an energy cut-off of 900 eV and a 1×1×1 k-mesh for a 4×3×2 super cell. The overall agreement is excellent and is supporting the use of the current methodology. The minor deviations for some of the optical phonon bands are attributed primarily to slight cell mismatches as a consequence of the full geometry optimizations performed in both codes.

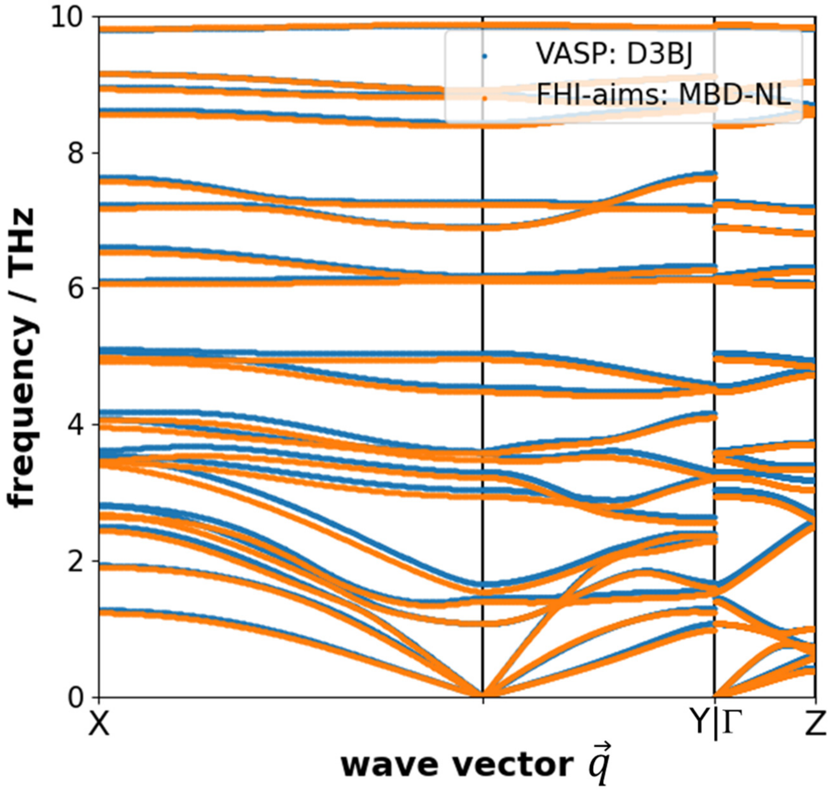

Figure S7: Low-frequency band structure of a-QA calculated with VASP and PBE+"D3(BJ)" (blue data points) and FHI-aims and PBE+"MBD-NL" (orange data points).

8. Super cell convergence:

We calculated the force constants for both the 4×3×2 (blue lines Figure S8) and the 6×4×2 (orange lines Figure S8) super cell with the cheaper "intermediate" basis sets to confirm that the 4×3×2 super cell is large enough to obtain converged vibrational properties of α-QA. Calculations using the "tight basis set" and a 6×4×2 supercell are not affordable. The phonon band structures for the different super cell dimension are overlaid in Figure S8 and presented for an increased number of high-symmetry paths according to ref. [13]. The root-mean-squared deviation between the two is 0.03 THz (1.1 cm^{-1}) for modes below 4.2 THz (band gap in Figure S8) and 0.02 THz (0.5 cm^{-1}) including all modes. Towards high-symmetry points of the Brillouin zone boundary, some bands exhibit deviations, due the commensurability of the 6×4×2 super cell dimension with all these points, which the 4×3×2 cell does not have.

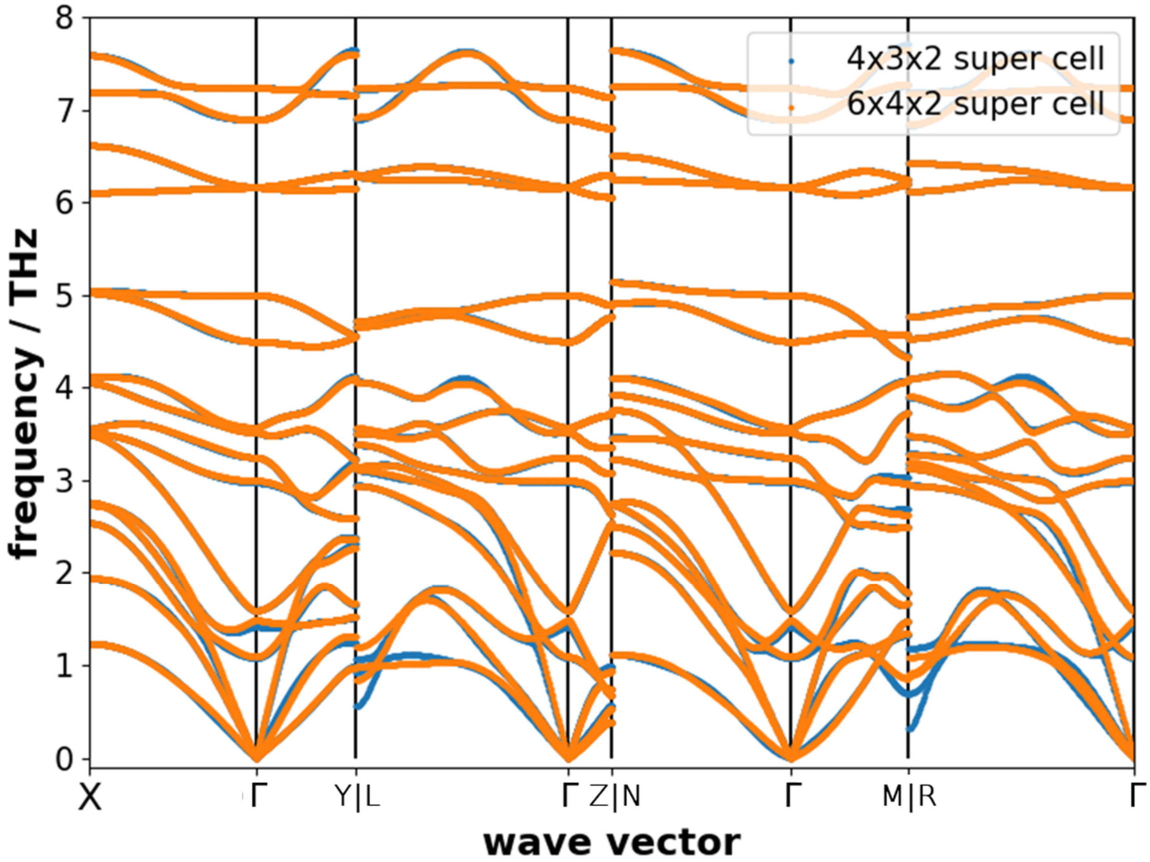

Figure S8: Phonon band structures calculated with 4×3×2 super cells (blue) and 6×4×2 (orange) supercells of α-QA for various high-symmetry paths in the 1st Brillouin zone, calculated with "intermediate" basis sets.

For the results presented in the main work we calculated the force constants for a 4×3×2 super cell using “tight” FHI-aims basis sets, which was 2.2 as expensive as for “intermediate” basis sets. These two calculations with different basis sets but the same super cell dimensions are overlaid in Figure S9 and the root-mean-squared deviation is 0.04 THz (1.4 cm^{-1}) for modes below 4.2 THz and 0.06 THz (2.1 cm^{-1}) including all modes. Difference here mostly originate from the unit cell parameters of the primitive cell, which slightly change when relaxing with “tight” settings and affect the Γ-modes as well.

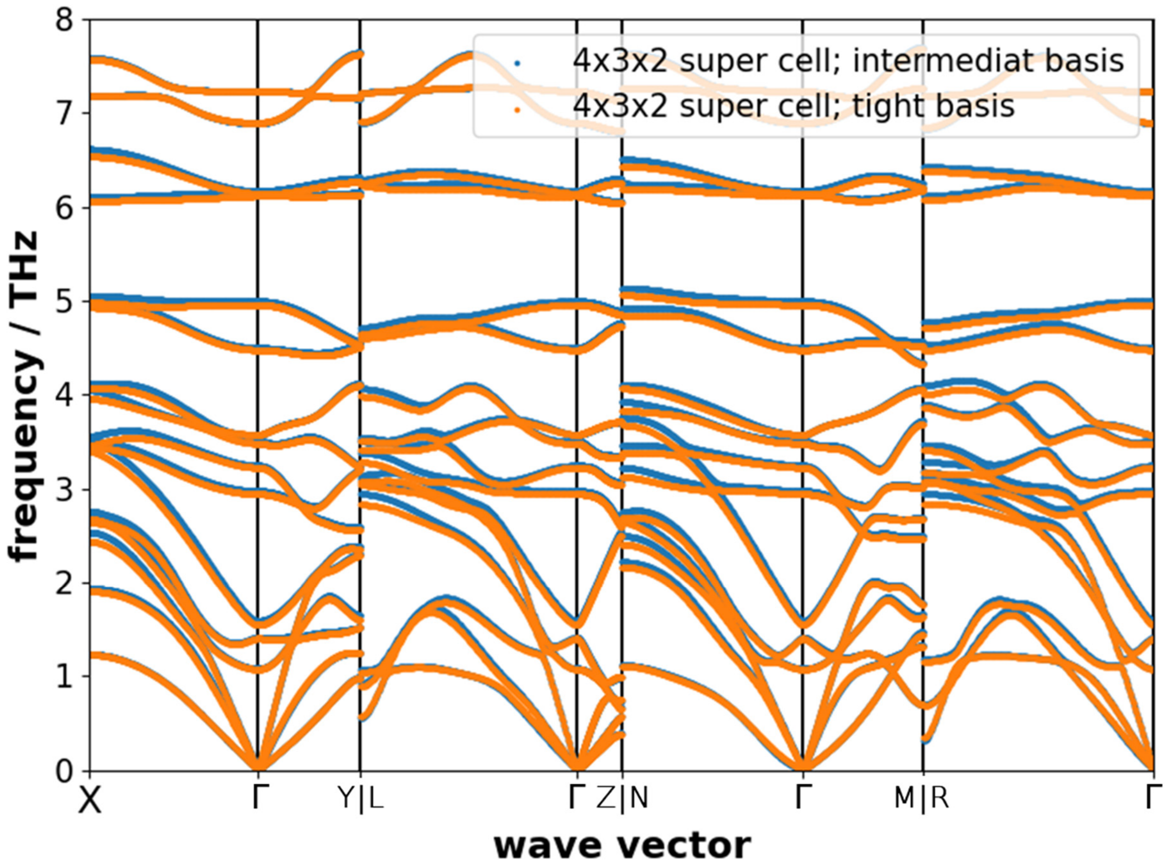

Figure S9: Phonon band structures of α-QA calculated with 4×3×2 super cells and an “intermediate” basis set (blue) and a “tight” basis set (orange) for each high-symmetry path of the 1st Brillouin zone.

The 6×4×2 calculation was performed with softer SCF convergence settings (1e-05 for charge density, 1e-06 for total energy and 1e-03 for sum of eigenvalues compared to the settings used in combination with tight basis set that are listed in Table S3). Overall, the differences of the band structures discussed in this section are rather small and do not influence the main findings of our work concerning acoustic band dispersions, avoided crossings, mode propagation, and the directional dependence of the vibrational properties.

9. The role of anharmonicities

When testing the impact of anharmonicities, one has to distinguish between effects related to thermal expansion, particularly large (temperature-induced) displacement amplitudes and the question, whether the performed simulations occur within the harmonic region of the potential-energy surface (PES). The thermal expansion could, e.g., be studied within the quasi-harmonic approximation, where the crystal volume is adjusted for thermal expansion, while still harmonic potential-energy surfaces are assumed. This is, however, beyond the scope of the present manuscript. Thus, such effects are not considered here and, in that sense, the data presented in the main manuscript mimic the situation close to 0K. Only towards the end of this section, we compare band structures obtained for experimental room temperature and DFT-optimized unit cells.

The test to what extent the calculations have been performed within the harmonic region of the PES, we performed a series of tests: As the first type of test, we employed different displacement amplitudes within the finite difference method. The default displacement, which is also used throughout the rest of this manuscript amounts to 0.01 Å and serves as a reference. Additionally, displacements of 0.1 Å, 0.05 Å, 0.02 Å, 0.001 Å were considered. The resulting displacement-amplitude dependent frequencies are shown in Figures S10 and S11 and the vibrational eigenvectors are compared in Figure S12. Table S7 summarizes the corresponding root mean square and maximum deviations. Overall, one sees that in the range between 0.001 Å to 0.02 Å there are no relevant differences in frequencies and eigenmodes. This shows that the reference displacement of 0.01 Å is well suited for sampling the PES around its minima in the harmonic regime. Only for the very large displacement amplitudes of 0.05 Å and 0.1 Å, there are increasing frequency shifts and the eigenvectors become less aligned to those of the 0.01 Å reference calculation. Notably, here modes below 30 THz shift towards lower frequencies, as can be seen in Figure S10, while above the gap around 30 THz, most modes shift to higher frequencies at larger displacements, as shown in Figure S11.

Table S7: Mean (RMSD) and maximum (MAE) frequency differences, given in wavenumbers (cm^{-1}), between tested and reference displacement amplitudes d, given in angstrom (Å), for modes below 10 THz (f<10 THz) and for all modes.

d / Å	RMSD / cm^{-1} (*f*<10 THz)	MAE / cm^{-1} (*f*<10 THz)	RMSD / cm^{-1}	MAE / cm^{-1}
0.001	0.0	0.2	0.2	0.6

0.020	0.1	0.5	0.5	1.5
0.050	0.9	3.7	4.0	11.4
0.100	4.0	18.0	17.2	46.1

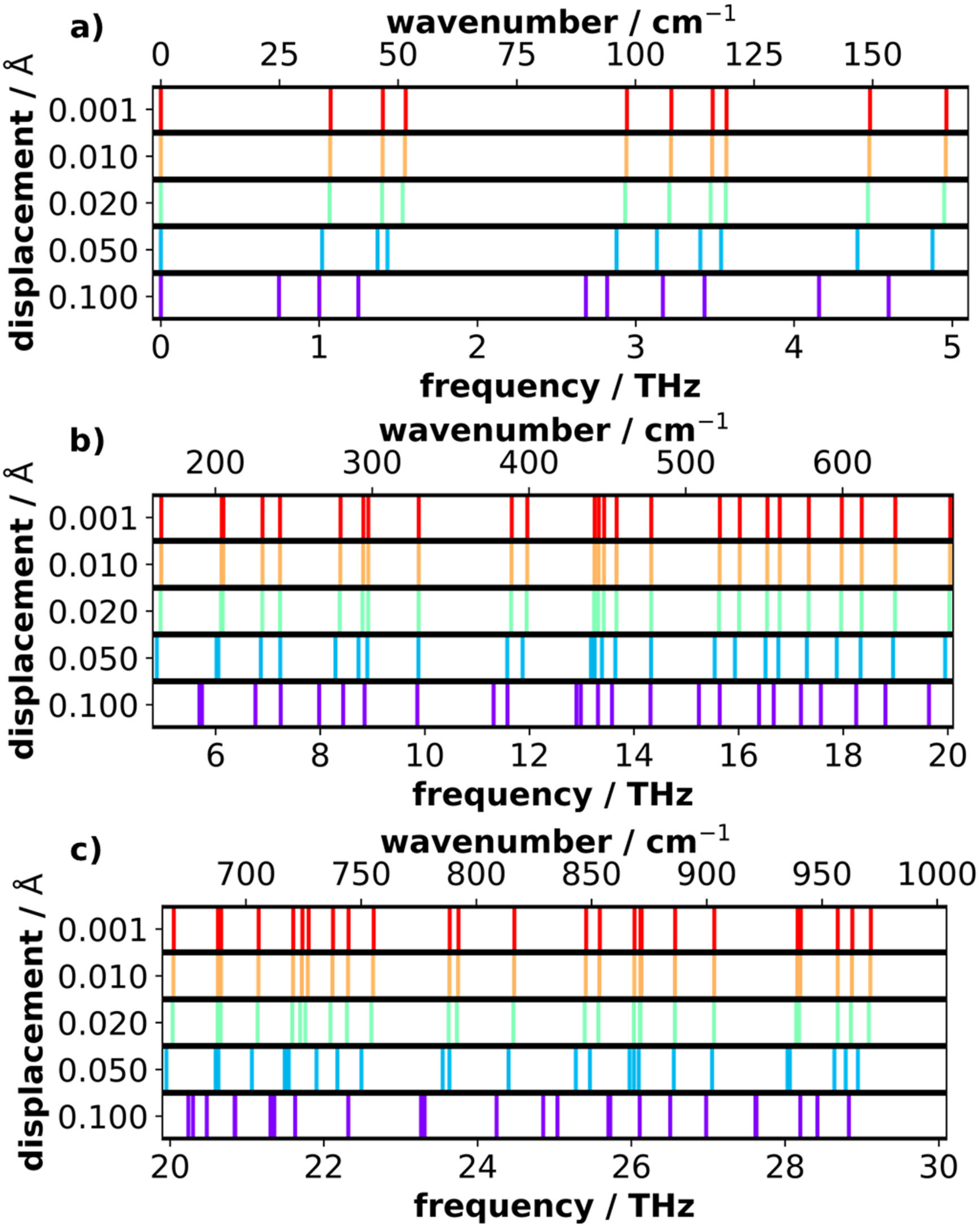

Figure S10:[illegible]-point frequencies for finite difference displacement amplitudes of 0.001 Å, 0.010 Å, 0.020 Å, 0.050 Å and 0.100 Å for consecutive frequency regions: 0 to 5 THz (a), 5 to 20 THz (b), and 20

to 30 THz (c). In these frequency regions, modes of larger displacements are shifted to lower frequencies.

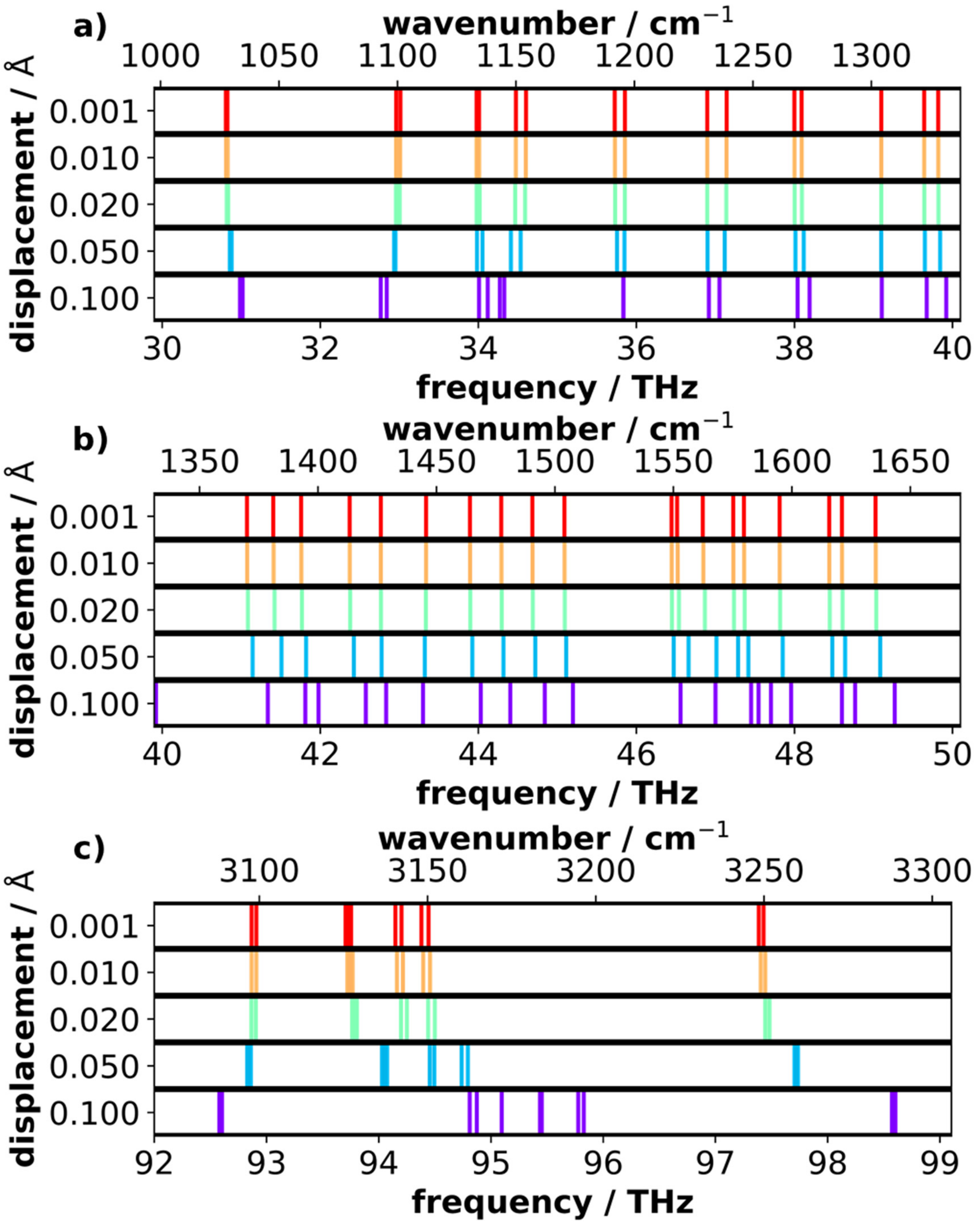

Figure S11: Γ-point frequencies for finite difference displacement amplitudes of 0.001 Å, 0.010 Å, 0.020 Å, 0.050 Å and 0.100 Å for consecutive frequency regions 30 to 40 THz (a), 40 to 50 THz (b), and 92 to 99 THz (c). In these frequency regions, most modes of larger displacements are shifted to higher frequencies.

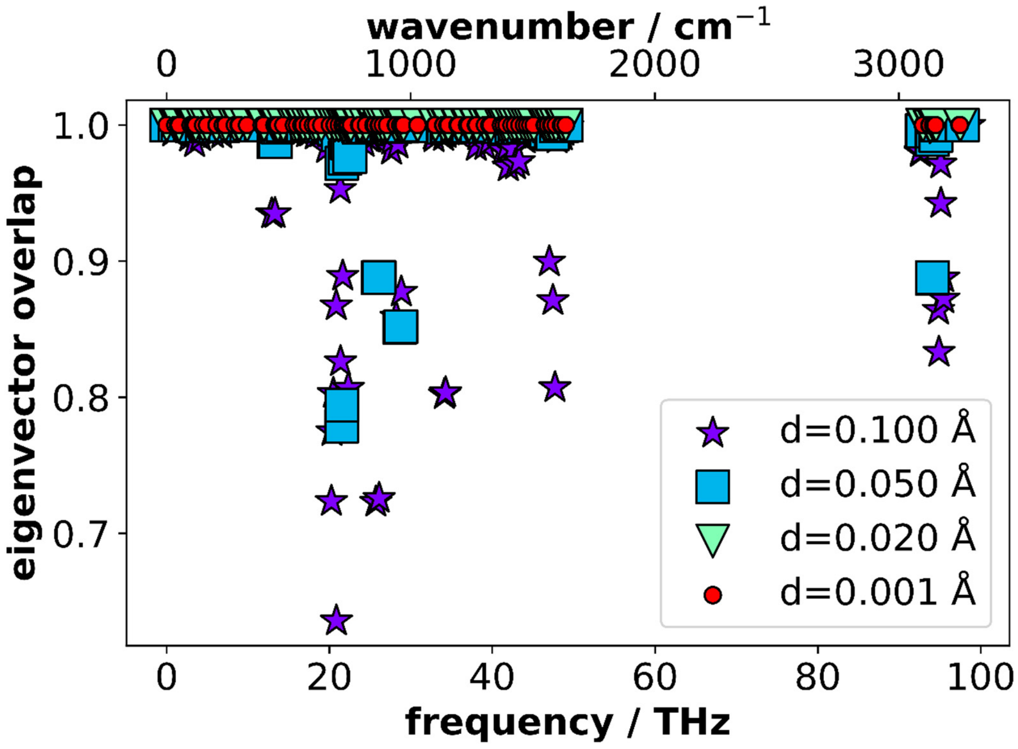

Figure S9: Eigenvector overlap between tested and reference displacement amplitude as a function of the vibrational frequency.

As a next step, the Γ-point frequencies of the fully optimized structure (V= 345 Å^3) were compared to those for the experimental unit cell (V= 348 Å^3) of α-QA. The results are plotted in Figure S13. There, the frequency differences are highlighted by the coloring of the data points. The frequencies of some higher-lying modes are shifted to higher values by up to 0.19 THz (red points), for most of the lower-frequency modes (below 10 THz), one observes a downwards shift, which is consistent with the mode-softening one would expect for a less densely packed structure. Most importantly, the data in Figure S13 show that at least the impact on the Γ-frequencies is comparably minor, suggesting that the low-temperature results presented here should also be representative of the room-temperature situation.

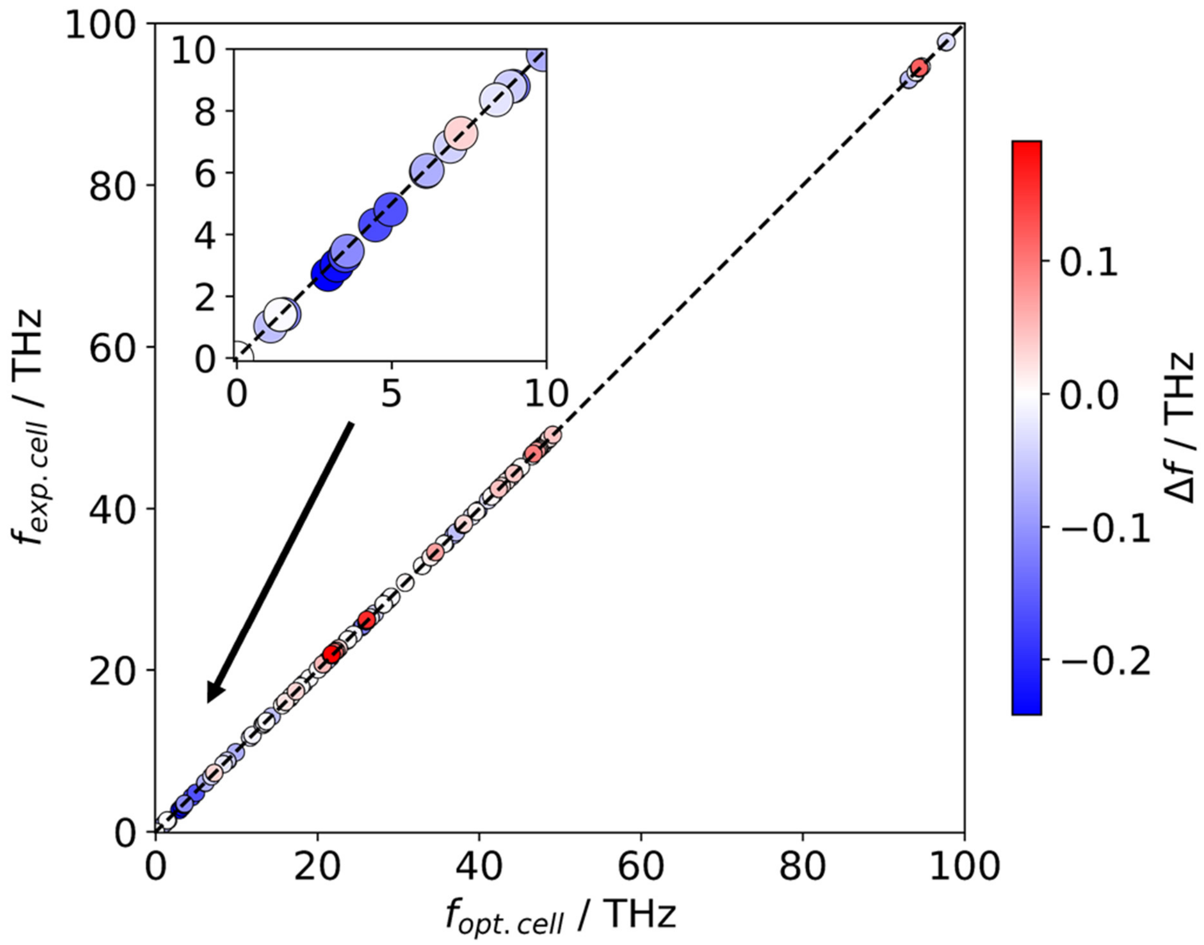

Figure S10: Γ-frequencies of the vibrations calculated for the experimental unit cell of α-QA as a function of the frequencies obtained for the fully optimized cell. The coloring highlights the magnitude of the differences. For blue data points the modes for the experimental cell are shifted to lower frequencies, while red points denote a shift to higher frequency values. The inset is a zoom into the region up to 10 THz.

Lastly, we analyzed possible anharmonicities of the low-frequency modes by displacing the structure along the mass-weighted eigenvectors of an eigenmode for several displacement distances Q and calculated the energies of the structures. The energies of these single point calculations are denoted by the blue dots in

Figure S11 and S15. These data points are compared to the local (parabolic) shapes of the PES derived from the harmonic frequencies of the eigenmodes. Said harmonic frequencies ω, that we get from the lattice dynamics calculation, can be transformed into an energy as a function of the eigen displacement distance in the following way:

$$E(Q) = \frac{\omega^2 \mu}{2} Q^2 \tag{S1}$$

with the normal mode coordinate Q, and the effective mass of the mode μ. $E(Q)$ is denoted by a solid orange line in

Figure S11 and S15. Its minimum is defined to be zero. This equation is akin to a term in the Hamiltonian of the quantum harmonic oscillator equation. It comes about by writing the Hamiltonian of phonons as independent one-dimensional harmonic oscillators. The effective mass μ is given by

$$\mu = \frac{\sum_i^N |\vec{e}_i|^2}{\sum_i^N m_i^{-1} |\vec{e}_i|^2} \tag{S2}$$

with the eigenvector $\vec{e}_i$, the mass of each atom m_i and the sum over all atoms i. For simplicity, we omitted the subscripts, that show the dependence on the band index and reciprocal lattice vector. These equations hold true for each band index and reciprocal lattice vector. In the following, we focus on bands 3-10 at the Γ-point. Bands 1-3 correspond to translations of the whole crystal. Of course, these translational modes yield no change in energy, since the system is translationally invariant. To illustrate this point we also show mode 3.

We propose to look at the differences between the explicit and harmonic calculation up to energies of 25 meV, (k_BT at room temperature). As can be seen in Figures S14 and S15, the explicitly calculated energies of displaced structures lie close to the harmonic energies of the corresponding eigenmodes, at least for energies up to 25 meV. Only modes 4 and 6 appear to have some slight anharmonicity below an energy of 25 meV. For mode 4, the displaced structure at Q=0.1 has a lower energy (by 0.09 meV) than the Q=0 structure. It appears, that the energy curve of the explicit calculation is shifted by Q=0.1, with respect to the curve obtained in the harmonic calculation. We attribute this to the relaxation based on maximum residual forces, which (for the chosen convergence criterion) cannot fully capture the minor differences in the molecular arrangement that this rotation mode entails.

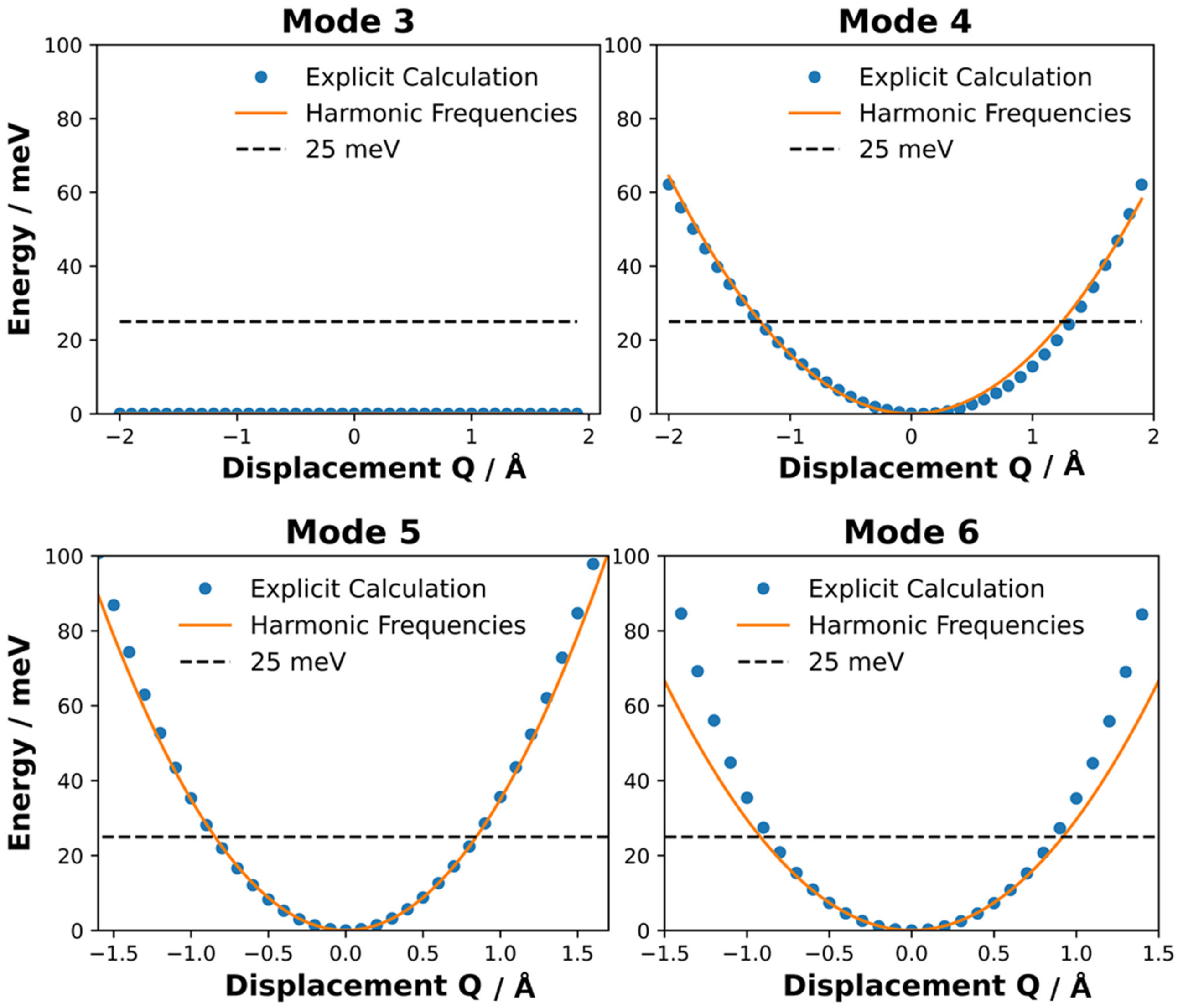

Figure S11: Energy (compared to equilibrium) versus eigenmode displacement Q for modes 3 to 6. The blue dots show the explicitly calculated energies, while the orange line show the parabolic potential energy surfaces derived from eigenfrequencies within the harmonic approximation. The dashed black line indicates the thermal energy at room temperature.

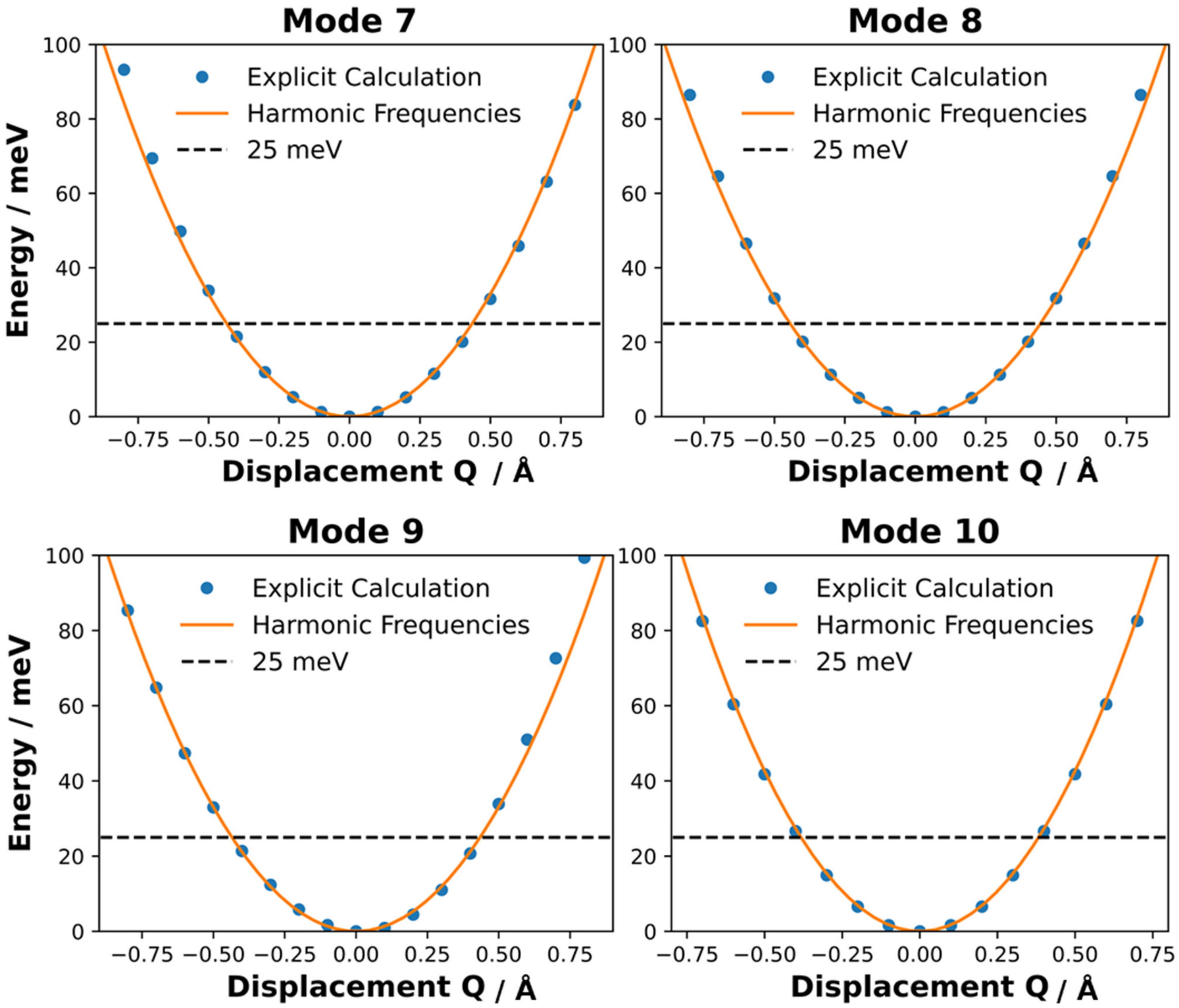

Figure S12: Energy (compared to equilibrium) versus eigenmode displacement Q for modes 7 to 10. The blue dots show the explicitly calculated energies, while the orange line show the parabolic potential energy surfaces derived from eigenfrequencies within the harmonic approximation. The dashed black line indicates the thermal energy at room temperature.

9. Analysis of the displacement types for the acoustic phonons of α-QA

In Figure S16 the band structure (i.e., the dependence of frequencies on $|\vec{q}|$) is plotted for the conventional band path along high-symmetry directions (Figure S16 a-c) and along the molecular axes (Figure S16 d-f), as elaborated in more detail in the main paper. The phonon bands are colored

according to their displacement character. Figures S16 highlights displacements in direction of the molecular plane normal in a) and d), the short axis in b) and e), and the long axis in c) and f).

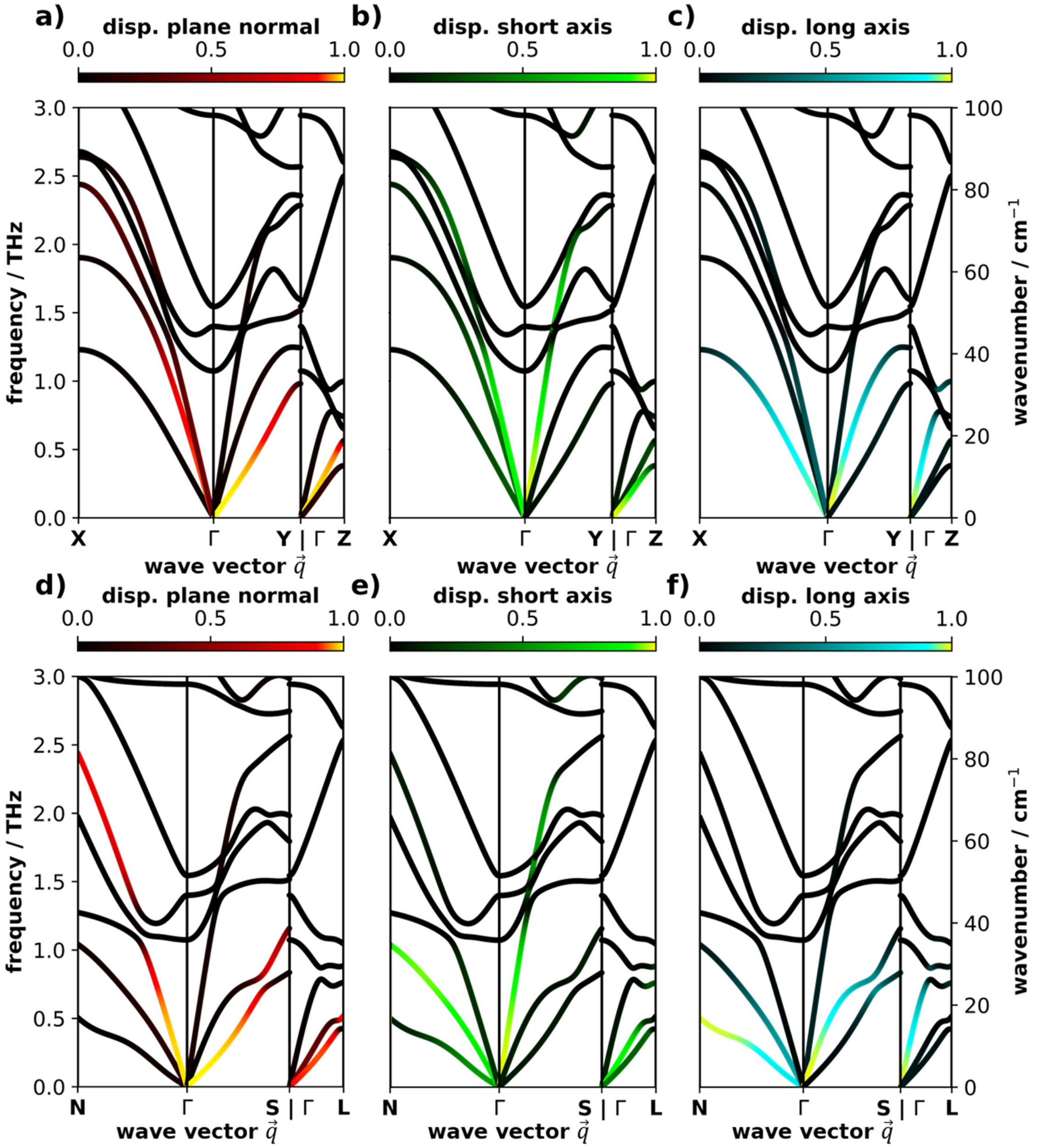

Figure S16: Phonon band structure of α-QA for wave vectors parallel to the high-symmetry paths (a-c) and parallel to the molecular axes (d-f). The coloring indicates how much the vibrational mode represents a translation in direction of the a,d) plane normal, b,e) the short axis, and c,f) the long axis of the molecule.

10. Avoided crossings and mode propagation along high-symmetry paths

The involved phonon band structure of α-QA necessitates close-up presentations of certain aspects, like avoided crossings and the propagation of specific vibrational modes throughout the Brillouin zone. Akin to the close-ups shown in Figures 4b-d in the main paper, such plots are presented in Figure S17. The phonon bands are again colored according to their mode participation ratios and to further elucidate the mode propagation towards the Brillouin zone boundary, the bands are assigned with their respective Γ-mode numbers. In Figure S17a the complex mode propagation of the acoustic (1, 2, 3), rotational (4, 5, 7) and lowest intramolecular modes (6, 8) from Γ to X can be followed by following similarly colored band segments. However, in Figure S17b the propagation and subsequential de-coupling of mode 7 and 9 (as discussed in section 4.4 in the main work) is more ambiguous and cannot be followed throughout the avoided crossings via the participation ratio, which can be rationalized by the severe change in the displacement character, when the long axis rotation and 2nd order out of plane bending modes are decoupled. At the Brillouin zone boundary, mode 7 has lost its bending character and mode 9 lost its rotational character.

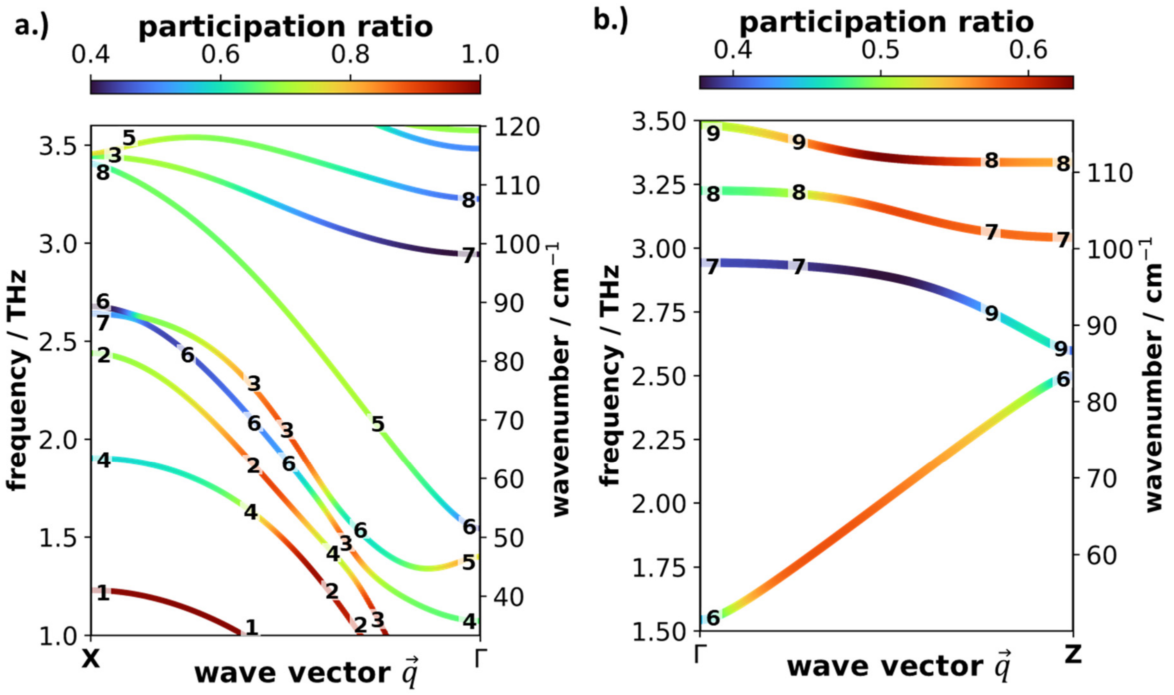

Figure S17: Close-ups of the phonon band structure of α-QA13 for a.) the XΓ path between 1 and 3.6 THz and b.) the ΓZ path between 1.5 and 3.5 THz, in units of THz on the left and wavenumbers on the right axis. The bands are colored according to their respective mode participation ratios. Please note the different ranges values of mode participation ratios for a.) and b.) in the color bars on top of the plots. Numbers on the bands label the mode character and refer to the labelling of Γ-modes in Figure 2a and Figure 4a.

11. Avoided crossings of the angular band structures

In the section about angular band structures we claim that the intersecting points between band 3 and band 2 are avoided crossings with gaps too narrow to see in Figure 6a. Therefore, Figure S18 shows a close up of the discussed region around the avoided crossing of the highest and second-highest acoustic bands. As indicated by the coloring of the data points, the longitudinality of the higher-frequency band decreases and that of the lower band increases towards the angle of $\vec{q}$ at the smallest gap. There, band 2 appears to be completely longitudinal and band 3 completely transversal, followed by a switch in band character.

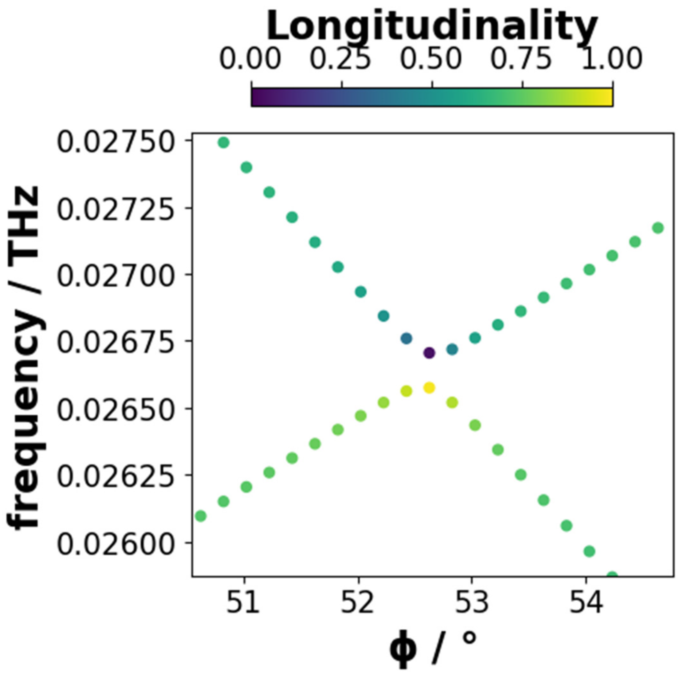

Figure S18: Close-up of an angular phonon band structure, i.e. the dependence of the vibrational frequency on the direction of $\vec{q}$, as presented in Figure 6a around an avoided crossing.

(1) Blum, V.; Gehrke, R.; Hanke, F.; Havu, P.; Havu, V.; Ren, X.; Reuter, K.; Scheffler, M. Ab Initio Molecular Simulations with Numeric Atom-Centered Orbitals. *Computer Physics Communications* **2009**, *180* (11), 2175–2196. https://doi.org/10.1016/j.cpc.2009.06.022.

(2) Blum, V.; Gehrke, R.; Hanke, F.; Havu, P.; Havu, V.; Ren, X.; Reuter, K.; Scheffler, M. Ab Initio Molecular Simulations with Numeric Atom-Centered Orbitals. *Computer Physics Communications* **2009**, *180* (11), 2175–2196. https://doi.org/10.1016/j.cpc.2009.06.022.

(3) F. Paulus, E.; J. Leusen, F. J.; U. Schmidt, M. Crystal Structures of Quinacridones. *CrystEngComm* **2007**, *9* (2), 131–143. https://doi.org/10.1039/B613059C.

(4) Perdew, J. P.; Ernzerhof, M.; Burke, K. Rationale for Mixing Exact Exchange with Density Functional Approximations. *J. Chem. Phys.* **1996**, *105* (22), 9982–9985. https://doi.org/10.1063/1.472933.

(5) Hermann, J.; Tkatchenko, A. Density Functional Model for van Der Waals Interactions: Unifying Many-Body Atomic Approaches with Nonlocal Functionals. *Phys. Rev. Lett.* **2020**, *124* (14), 146401. https://doi.org/10.1103/PhysRevLett.124.146401.

(6) Řezáč, J.; Riley, K. E.; Hobza, P. S66: A Well-Balanced Database of Benchmark Interaction Energies Relevant to Biomolecular Structures. *J. Chem. Theory Comput.* **2011**, *7* (8), 2427–2438. https://doi.org/10.1021/ct2002946.

(7) Reilly, A. M.; Tkatchenko, A. Understanding the Role of Vibrations, Exact Exchange, and Many-Body van Der Waals Interactions in the Cohesive Properties of Molecular Crystals. *J. Chem. Phys.* **2013**, *139* (2), 024705. https://doi.org/10.1063/1.4812819.

(8) *Toward a Reliable Description of the Lattice Vibrations in Organic Molecular Crystals: The Impact of van der Waals Interactions | Journal of Chemical Theory and Computation*. https://pubs.acs.org/doi/abs/10.1021/acs.jctc.8b00484 (accessed 2022-04-20).

(9) Kamencek, T.; Wieser, S.; Kojima, H.; Bedoya-Martínez, N.; Dürholt, J. P.; Schmid, R.; Zojer, E. Evaluating Computational Shortcuts in Supercell-Based Phonon Calculations of Molecular Crystals: The Instructive Case of Naphthalene. *J. Chem. Theory Comput.* **2020**, *16* (4), 2716–2735. https://doi.org/10.1021/acs.jctc.0c00119.

(10) Grimme, S.; Ehrlich, S.; Goerigk, L. Effect of the Damping Function in Dispersion Corrected Density Functional Theory. *Journal of Computational Chemistry* **2011**, *32* (7), 1456–1465. https://doi.org/10.1002/jcc.21759.

(11) Moellmann, J.; Grimme, S. DFT-D3 Study of Some Molecular Crystals. *J. Phys. Chem. C* **2014**, *118* (14), 7615–7621. https://doi.org/10.1021/jp501237c.

(12) Bučko, T.; Lebègue, S.; Gould, T.; Ángyán, J. G. Many-Body Dispersion Corrections for Periodic Systems: An Efficient Reciprocal Space Implementation. *J. Phys.: Condens. Matter* **2016**, *28* (4), 045201. https://doi.org/10.1088/0953-8984/28/4/045201.

(13) Setyawan, W.; Curtarolo, S. High-Throughput Electronic Band Structure Calculations: Challenges and Tools. *Computational Materials Science* **2010**, *49* (2), 299–312. https://doi.org/10.1016/J.Commatsci.2010.05.010.
